# Hypertranscription of rDNA Responsible for Nucleolar Remodelling is a Doorman for Acquiring Pluripotency

**DOI:** 10.1111/cpr.70052

**Published:** 2025-05-04

**Authors:** Yuchen Sun, Xinglin Hu, Xingwei Huang, Wenyao Zhou, Shubing Lan, Hui Zhang, Guangming Wu, Lei Lei

**Affiliations:** ^1^ Department of Histology and Embryology, Basic Medical Science College Harbin Medical University Harbin Heilongjiang P. R. China; ^2^ Guangzhou Laboratory Guangzhou Guangdong China

**Keywords:** chromatin accessibility, iPSC reprogramming, nucleoli, rDNA

## Abstract

Ribosome biogenesis occurs within the nucleolus, with the initial step being the transcription of ribosomal DNA (rDNA). Although rDNA transcription is limited in somatic cells, it is more active in stem cells. Nevertheless, the mechanisms involved in somatic cell reprogramming remain elusive. Both somatic and stem cell nucleoli exhibit a reticular structure. However, under the electron microscope, we identified an intermediate nucleolar state during reprogramming. This state underwent changes characterised by rDNA hypertranscription, resulting in an enlarged nucleolus, enhanced activity of nucleolus organiser regions (NORs), and a transition from the reticular nucleolar type to an intermediate state of reprogramming, whose three liquid phase boundaries are blurred. Our research revealed that Oct4 was directly targeted to the rDNA enhancer region, promoting its hypertranscription and nucleolar enlargement during reprogramming. Using rDNA transcriptional inhibitors, we proved that nucleolar remodelling and subsequent reprogramming are halted by inhibiting rDNA transcription. But why could rDNA transcriptional activity influence reprogramming? Our findings elucidate that the active nucleoli have the capability to release perinucleolar heterochromatin. By joint analysis of Assay for Transposase‐Accessible Chromatin with high throughput sequencing (ATAC‐seq) and RNA‐seq, we have characterised the perinucleolar chromatin released by the nucleolus in a reprogramming intermediate state. The released chromatin mainly impacted mesenchymal‐to‐epithelial transition (MET)‐related genes. MET is a stage of silencing of mesenchymal genes, accompanied by the activation of epithelial genes. Concurrently, the morphology of mouse embryonic fibroblast cells (MEFs) transitions from elongated spindle‐shaped cells to short roundish forms, exhibiting a propensity to cluster together. MET was considered an early event in reprogramming; our findings suggested that nucleolar remodelling occurred before MET.

## Graphical abstract

Active nucleoli have the capability to release perinuclear heterochromatin, which is a novel event of iPSC reprogramming.

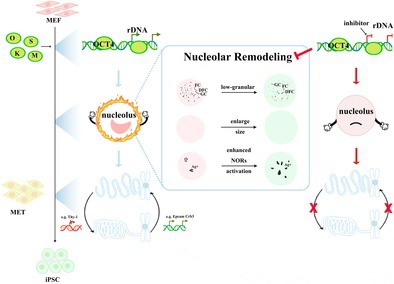

## 1 Introduction

The nucleolus, a membraneless organelle located in the interphase nucleus, is a round liquid–liquid phase separation region that forms around NORs [1]. The ultrastructure of the nucleolus consists of three coexisting liquid phases to perform ribosome biogenesis: fibre centre (FC), dense fibre centre (DFC) and granular centre (GC). The FC region, housing the rDNA loci, is considered the central and essential region of the nucleolus. Surrounding the FC is the DFC, where pre‐rRNA transcribed from the FC is processed and cleaved into the mature 18S, 5.8S and 28S rRNAs. GC serves as the peripheral region of the nucleolus, facilitating the assembly and maturation of pre‐ribosomal particles into functional ribosomes [2]. Fibrous reticular nucleoli with three distinct liquidphases are commonly observed in stem cells and somatic cells. However, specialised nucleoli emerged during cellular fate transitions.

In mouse oocyte maturation, the reticular nucleoli transform into nucleolus‐like bodies (NLBs) in fully grown oocytes [3]. After fertilisation, a transient inactive state of nucleoli, known as nucleolus precursor bodies (NPBs), occurs in early embryos, characterised by the absence of rRNA transcriptional activity [4]. During early embryonic development, NPBs support chromatin remodelling around centromeres and centrosomes, ensuring proper chromosome segregation and playing a crucial role in zygotic genome activation (ZGA) [5]. Following ZGA, which is the 2‐cell stage in the mouse embryo, NPBs gradually mature into reticular nucleoli. Matured reticular nucleoli sequester the Dux gene locus within the perinucleolar heterochromatin compartment, suppressing the Dux gene, thereby facilitating the exit from the 2‐cell state and transition from totipotency to pluripotency [3, 6].

The nucleolus undergoes similar changes during the process of somatic cell reprogramming to an embryonic state. In our previous studies, we observed that during somatic cell nuclear transfer (SCNT), somatic nucleoli could be directly remodelled into NPBs and subsequently transformed into reticular nucleoli in the embryos [7]. Moreover, the process of nucleolar remodelling affects the blastocyst rate of SCNT embryos [8]. As two distinct somatic cell reprogramming methodologies, it is widely accepted that SCNT reprograms somatic cells into a totipotent state, whereas induced pluripotent stem cell (iPSC) technology reprograms somatic cells into a pluripotent stem cell state [9].

iPSCs exhibit enlarged nucleoli and increased levels of rDNA transcription compared to their pre‐reprogramming counterparts [10, 11]. The existence of intermediate nucleolar states during the process of iPSC reprogramming remains an unresolved question. In February of 2024, Jin Zhang et al. discovered that the reprogramming factor LIN28A regulates the phase structure of nucleoli, and the presence or absence of its IDR domain determines the efficiency of generating an iPSC line [12]. Are there other reprogramming factors capable of enhancing rDNA transcription? In the process of iPSC reprogramming, at what stage do changes in the nucleolus occur? Furthermore, the precise impacts of fluctuations in rDNA transcriptional activity and nucleolar dynamics on the acquisition of stem cell characteristics during somatic reprogramming are still unclear. In the process of cellular transition to totipotency, dynamic changes in the nucleolus lead to the expression of DUX [6, 13], but what specific genes are released during the transition to pluripotency in the context of iPSC reprogramming? These questions remain unexamined.

In the present study, we first explored the increased expression of nucleolar function‐related genes during the initial 2 days of iPSC reprogramming through RNA sequencing (RNA‐seq) and scRNA‐seq analysis. Subsequently, we monitored various aspects of the nucleolus throughout this period. Nucleolar activity was confirmed to be robust, evidenced by its size, morphology, rDNA transcriptional activity and ultrastructure. To investigate how the activation of the nucleolus in the early stage of iPSC reprogramming is achieved, we verified that the reprogramming factor OCT4 directly targets rDNA using chromatin immunoprecipitation followed by quantitative polymerase chain reaction (ChIP‐qPCR). This further confirmed that rDNA transcriptional activation induces nucleolar remodelling through multiple rDNA transcription inhibitors. Finally, by jointly analysing ATAC and RNA‐seq after inhibiting rDNA transcription, we characterised the perinucleolar chromatin released by the nucleolus in a reprogramming intermediate state, which primarily affected genes associated with the MET. The process of MET is a critical phase within the reprogramming of iPSC, wherein there is a decline of transcriptional activity for mesenchymal‐specific genes, concomitant with the increase of expression of epithelial lineage genes. During this period, the cytoarchitectural morphology of MEFs undergoes a transformation from an elongated spindle‐like configuration to a short roundish structure, with an increased tendency towards cellular aggregation [14]. This investigation highlights the activation of rDNA induced by OCT4 in the first 2 days, with resulting nucleolar remodelling as a pivotal and initial process in iPSC reprogramming and influencing chromatin accessibility. Our study demonstrates the involvement of nucleolar mechanisms in iPSC reprogramming and emphasises the significance of rDNA as a key responder in cell fate transition.

## 2 Results

### 2.1 Activation of Nucleolar Function‐Related Genes Was Mainly in the Initial 2 Days of iPSC Reprogramming

**FIGURE 1:**
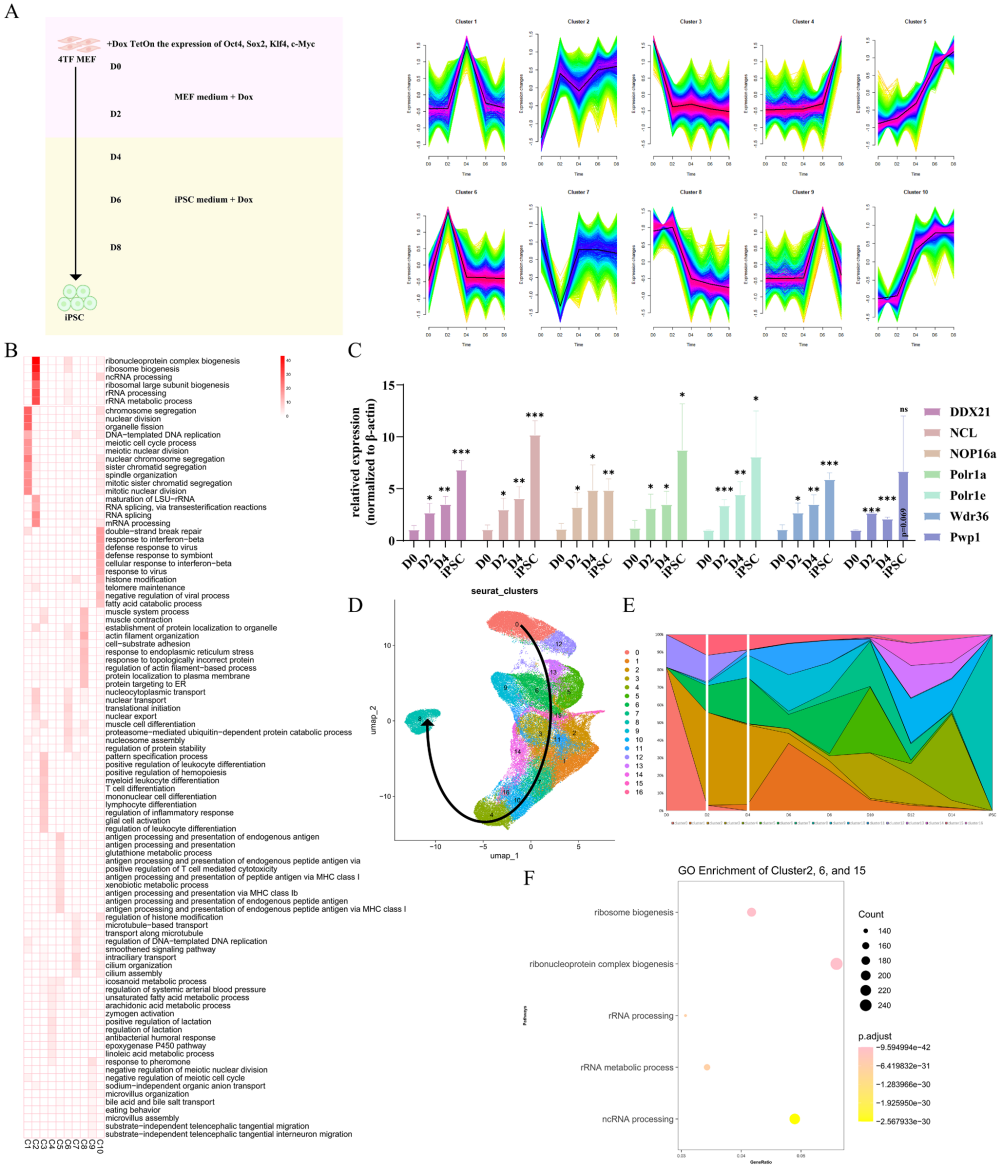
Activation of nucleolar function‐related genes was mainly in the first 2 days of iPSC reprogramming. (A) Left: Schematic representation of the process of iPSC reprogramming. Right: Temporal clustering of RNA‐seq data of cells in iPSC reprogramming. Fuzzy c‐means clustering identified 10 distinct temporal patterns of gene expression during iPSC reprogramming. The *X*‐axis represents four points in time for iPSC reprogramming (D0, D2, D4, D6, D8), whereas the *Y*‐axis represents log2‐transformed, normalised intensity ratios at each stage. The black line in each cluster is the *Z*‐line, which represents the overall expression trend. The genes in clusters can be found in Table S2. (B) The heat map shows the –log10 transformed significance value of the top 10 biological processes terms in each of the 10 clusters. (C) RT‐qPCR analysis of nucleolar functional related genes DDX21, NCL, Nop16, Polr1a, Polr1e, Wdr36 and Pwp1 in the early stage of iPSCs reprogramming. *p < 0.05, **p < 0.01, ***p < 0.001 (*n* = 3, Student's *t*‐test, mean ± SD). (D) UMAP of single‐cell RNA‐seq labelled by characteristics of cells. Reanalysis of data from GSE242424. (E) The accumulation area map shows the distribution of clusters at different days of reprogramming. The size of the colour block area reflects the proportion of each cell type within the corresponding cluster. From the *Y*‐axis to the first line, the proportion of orange colour blocks is nearly 0 on day D0. However, by D2, the yellow colour blocks represent more than 50%, indicating that most cells have transitioned to this cluster's cellular identity. From the *Y*‐axis to the second line, the proportion of purple decreases from 20% to nearly 0 by D4, suggesting that the cells in this cluster have completely transitioned to other identities. (F) GO and KEGG enrichment analysis demonstrated that the upregulated marker genes of clusters 2, 6 and 15 mainly enriched in ribosome biogenesis.

The 4F2A transgenic mouse model is genetically modified to harbour a doxycycline (Dox)‐inducible expression cassette encoding the pluripotency‐associated transcription factors OCT4, SOX2, KLF4 and c‐MYC. These mice are further engineered to incorporate a col1a1 promoter‐driven TetOn regulatory system for the conditional expression of the aforementioned factors. For our experimental purposes, fibroblasts were isolated from E13.5 embryos of the 4F2A strain. After the cells were successfully cultured, adding Dox triggered the reprogramming process. This process enabled the cell population to regain pluripotent characteristics (Figure 1A, left).

First, we want to identify the most important period of iPSC reprogramming. To achieve this, we collected mRNA samples at five distinct time intervals (D0, D2, D4, D6 and D8) during the iPSC reprogramming process for subsequent RNA‐seq analysis, aiming to profile the global gene expression dynamics. Unsupervised hierarchical clustering analysis of gene expression profiles revealed that D6 and D8 samples clustered together in the same branch, suggesting minimal differences between them. Similarly, D4 grouped with D6 and D8 in the same major branch, indicating a high degree of similarity among these stages, which implied that gene expression changes during the later stages of reprogramming were relatively subtle. In contrast, D0 and D2 were placed in a separate major branch, suggesting that the changes occurring before Day 4 are more pronounced (Figure S1A). This clustering trend was also confirmed in principal component analysis (PCA), where samples were grouped accordingly. The projection of each point on the *X*‐axis highlighted significant differences between D0 and D2, as well as between D2 and D4, which were further reflected in the projection on the *Y*‐axis (Figure S1B).

Additionally, we conducted a differential expression gene (DEG) analysis across four time points (D2 vs. D0, D4 vs. D2, D6 vs. D4, D8 vs. D6) during the iPSCs reprogramming process. The number of DEGs was identified with a significance level of *p* < 0.05. The greater the number of DEGs and the higher the fold change of these genes, the more significant the differences between the two groups of samples. The number of DEGs decreased progressively from almost 10,000 in D2 versus D0 and D4 versus D2 to 6851 in D6 versus D4 and subsequently to less than 5000 in D8 versus D6. The observed differences in gene expression among D0, D2 and D4 were significantly larger based on the number and magnitude of DEGs (Figure S1C). Notably, the analysis indicated significant alterations in gene expression patterns between D0–2 and D2–4 during iPSCs reprogramming, suggesting a more pronounced shift in gene expression prior to reprogramming at D4 compared with the changes observed in D4–6 and D6–8.

The process of iPSC reprogramming encompasses dynamic and continuous alternations in global gene expression profiles. By employing the fuzzy c‐means algorithm, a temporal clustering method, we were able to discern 10 unique expression kinetics patterns throughout the reprogramming process. The *Z*‐line (a black line positioned centrally within each cluster) represented the temporal trajectory of gene expression variations during the reprogramming process. The colour signifies the probability of the expression trends within that cluster aligning with the *Z*‐line. Notably, clusters 2, 10, 5 and 4 exhibited the upregulated genes at D0–2, D2–4, D4–6 and D6–8 stages during iPSC reprogramming progress, respectively. Conversely, clusters 3 and 8 showed the downregulated genes at specific time points, namely D0–2 and D2–4, respectively (Figure 1A, right panel and Figure S1G).

Further analysis was conducted by subjecting the genes within each cluster to gene ontology (GO) analysis, focusing on biological process (BP). The results revealed that the upregulated genes in the D0–2 stage (cluster 2) primarily functioned in ribosome biogenesis, reflecting nucleolar functionality (Figure 1B). In contrast, the upregulated genes in the D2–4 stage (cluster 10) were predominantly associated with histone modification and innate immunity (Figure 1B). The downregulated genes in the D0–2 stage (cluster 3) were implicated in muscle cell differentiation, pattern specification process, myeloid leukocyte differentiation and lineage specification. Meanwhile, the downregulated genes in the D2–4 stage (cluster 8) were involved in cell substrate adhesion and protein localization to plasma membrane, processes crucial for cell aggregation and epithelial‐like morphological transitions (Figure 1B) [12]. These results indicated that, along with the well‐documented reprogramming‐associated alterations in somatic genes, chromatin remodelling and MET, the upregulation of ribosome biogenesis genes (cluster 2) is of particular interest as a potentially unexplored early reprogramming event.

We further confirmed that the expression of ribosome biogenesis‐associated genes increased overall from D0 to D2 (Figure S1D). Intriguingly, rDNA transcription‐related genes (GO#0042790) were predominantly observed in clusters displaying elevated kinetic profiles (clusters 2, 5 and 10) (Figure S1E). To illustrate, the gene Polr1h demonstrates a significantly higher probability (approximately 0.8) of being assigned to cluster C10 compared to other clusters, classifying Polr1h within this specific group. Notably, genes within cluster C10 exhibit a characteristic expression pattern, showing marked upregulation during Days 2–4 of the reprogramming process (Figure S1E). To concretely verify the expression dynamics of rDNA transcription‐related genes, the transcripts per kilobase of exon model per million mapped reads (TPM) values exhibited an upregulated trend in the expression of ribosome biogenesis‐related genes, including DDX21, Wdr36, Pwp1, NCL, NOP16, Polr1a and Polr1e (Figure S1F). Furthermore, the increased expression of these genes at the initial stage of reprogramming was confirmed through RT‐qPCR analysis (Figure 1C).

Additionally, the validation of our findings was accomplished through temporal clustering of RNA‐seq data pertaining to OSKM reprogramming, sourced from an independent research collective [15] (Figure S2A). Cluster 3 exhibited the steepest increase in gene expression during the D0–2 time frame, whereas cluster 1 showed significant upregulation primarily between D0 and D4. Subsequently, GO and KEGG enrichment analysis were performed on these two clusters, respectively (Figure S2B–E). The genes within both clusters exhibited enrichment in pathways associated with ribosome biosynthesis and neurodegenerative diseases. A previous study has established that mutations in genes involved in rDNA transcription serve as pathogenic factors in neurological degenerative disorders [16]. Subsequently, a temporal clustering analysis was conducted on data derived from an alternative iPSC reprogramming system employing SKM factors (Figure S3A). GO and KEGG analyses were performed on clusters 4 and 5, which showed an intense increase in global gene expression at D0–2 (Figure S3B–E).

In this study, we also reanalyzed the publicly available single‐cell RNA‐seq data of human fibroblasts across various reprogramming states [17]. Annotated cells were categorised into 16 clusters, with cells from Days 0–14 exhibiting a continuous change in reprogramming states (Figure 1D and Figure S1H). The accumulation area map illustrates the proportional changes of different cell clusters over time. Distinct populations of cells at different stages of reprogramming were identified, with clusters 2, 6 and 15 emerging on the second day of reprogramming (Figure 1E). GO analysis of upregulated marker genes within these clusters revealed significant enrichment in all processes associated with ribosomal biogenesis (Figure 1F). Hence, the upregulation of genes related to nucleolar function during the initial reprogramming stage is a common characteristic observed across species and reprogramming methodologies.

Through clustering analysis of nucleolar function‐related genes, we successfully delineated the timeframe of nucleolar alterations as occurring within the first 4 days of iPSCs reprogramming. Notably, D2 emerged as a pivotal juncture in the process of nucleolar remodelling. Building upon this discovery, our study further delves into the morphological and functional aspects of the nucleolus at D2 and D4 during iPSCs reprogramming, aiming to elucidate the underlying mechanisms responsible for the significant alterations observed in nucleolar activity during this period.

### 2.2 Dynamical Changes of Nucleolar Morphology and Structure–Nucleolar Remodelling

In this section, we investigated the process of nucleolar remodelling through three key perspectives: nucleolar relative size, active NORs and nucleolar ultrastructure. Firstly, we conducted immunofluorescent staining of nucleoli with antibodies targeting five nucleolar proteins: UBF, GNL3, FBL, B23 and NCL. The labelling and statistical analysis of these nucleolus proteins were utilised to minimise potential discrepancies among individual nucleolar proteins. The functions of these five proteins and their homologues are as follows. UBF is a component of the rDNA transcription initiation complex and plays a crucial role in ribosome biogenesis. The binding of UBF serves as an indicator of active rDNA loci, making it a recognised marker of NORs [17]. GNL3, also known as nucleostemin, typically increases during tissue regeneration following injury [18], primarily maintaining genome stability within the nucleolus [19]. In budding yeast, GNL3 is referred to as Nug1, acting as a GTPase associated with nuclear 60S pre‐ribosomes and necessary for the export of 60S ribosomal subunits from the nucleus [20]. Besides the function of ribosome biogenesis regulator, GNL3 has the ability to interact with RAD51 for DNA damage repair and with TRF1 to address telomere damage [21, 22]. FBL contains an intrinsically disordered region (IDR) domain rich in glycine–arginine (GAR) residues and a methyltransferase domain (MD) [23], implicated in the processing and cleavage of pre‐rRNA [2]. Yeast Nop1 is the homologue of FBL in mice, serving as the MD of C/D snoRNPs complex and required for the processing of pre‐18S rRNA [24]. NPM1, a crucial and abundant protein found in the nucleolus, primarily localises to the GC region and contributes to nucleolar structure formation [25]. In addition to its function as a histone chaperone protein, NPM1 is involved in rDNA transcription and ribosome biogenesis within the nucleolus [26]. NCL (Nsr1 in yeast), a eukaryotic nucleolar phosphoprotein, is engaged in the synthesis and maturation of ribosomes [27].

**FIGURE 2:**
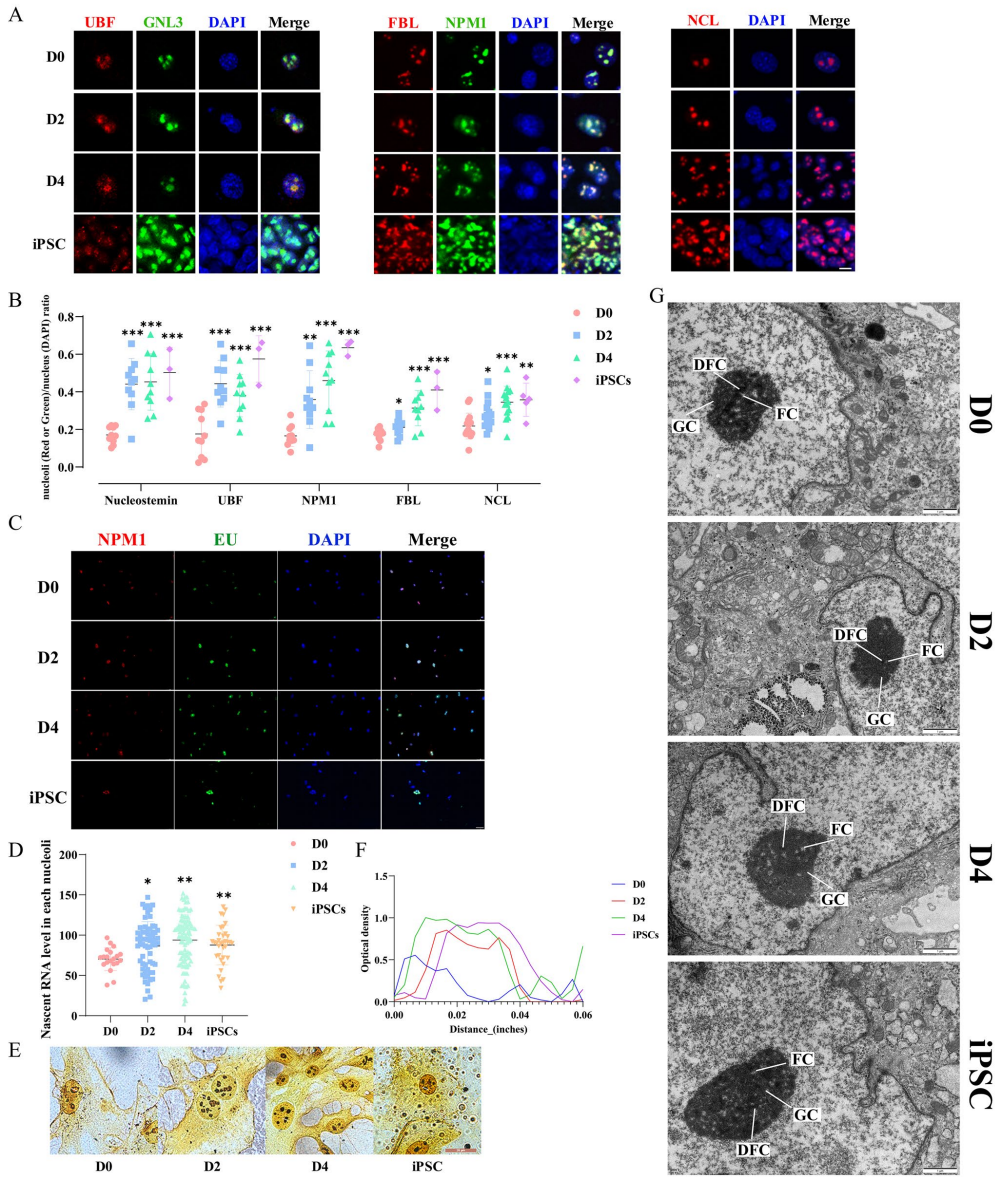
Dynamical changes of nucleolar morphology and structure—nucleolar remodelling. (A) Immunofluorescence of nucleolar proteins UBF, GNL3, FBL, NPM1 and NCL in the early stage of iPSCs reprogramming (D0, D2, D4) and iPSC. UBF serves as an indicator of active rDNA loci. GNL3 is necessary for the export of 60S ribosomal subunits from the nucleus. FBL is implicated in the processing and cleavage of pre‐rRNA. NPM1 contributes to nucleolar structure formation. NCL is engaged in the synthesis and maturation of ribosomes. Scale bar, 10 μm. (B) Analysing the area ratio of five nucleolar proteins to DAPI in (A), respectively. Each dot represents a nucleolus in D0, D2 and D4 or a whole clone of iPSCs. *p < 0.05, **p < 0.01, ***p < 0.001 (*n* = 10 in D0, 2, 4, *n* = 3 of iPSC clones, Student's *t*‐test, mean ± SD). (C, D) 5‐EU staining and immunofluorescence of NPM1 in the early stage of iPSCs reprogramming (D2, D4); D0 is the uninduced stage and iPSCs is the reprogramming finished stage. Scale bar, 50 μm. The (D) diagram is a statistic of the fluorescence intensity in each nucleolus in the (C) diagram. (E, F) The silver staining of NORs in the early stage of iPSCs reprogramming (D0, D2, D4) and iPSC (E), and analysis of silver stain particle density by optical density (F). As indicated in the figure, an increase in the number of black silver–stained particles within the NOR correlates positively with heightened transcriptional activity within the NOR. Scale bars, 20 μm. (G) The nucleolar ultrastructure in the early stage of iPSCs reprogramming (D0, D2, D4) and iPSC was observed by electron microscopy. The deeper the colour, the higher the electron density. The GC are the black regions within the nucleolus, with DFC in grey and FC in light grey. Scale bar, 1 μm.

Here, we estimated the relative nucleolar size by quantifying the area occupied by nucleolar proteins, adjusted for the DAPI‐stained area, to account for potential variations in cell size that could impact nucleolar size analysis. The immunofluorescent signals of the nucleolar proteins exhibited strong colocalization with rapidly expanding nucleoli at the early stage of reprogramming, as illustrated in Figure 2A. The relative nucleolar size significantly increased from approximately 0.2 at D0 to about 0.5 in iPSCs, with both D2 and D4 of the reprogramming process displaying nucleolar relative size exceeding 0.4, approaching the value observed in iPSCs (Figure 2B). Detailed information regarding nucleolar area ratios is available in Table S3.

Approximately 70% of nascent transcripts are produced in the nucleolus, allowing for the monitoring of nucleolar rRNA biogenesis by using 5‐ethynyl uridine (5‐EU) [28]. Through comprehensive 5‐EU staining analysis of whole cells, we observed a progressive increase in nascent RNA levels throughout the reprogramming process (Figure S4B,C). To determine whether this elevation was primarily attributed to the 70% rRNA component, we performed co‐staining experiments using NPM1 as a nucleolar marker in conjunction with EU labelling. By the co‐staining with NPM1, gradual enhancement of the 5‐EU signal per nucleolus during reprogramming indicated an increase in nucleolar transcriptional activity (Figure 2C,D). Moreover, NORs are the sites of nucleolus formation and are located within the secondary constriction regions of specific chromosomes in the nucleus [29]. To further assess this activity, NORs were visualised by staining with silver nitrate (Figure 2E). The presence of darker silver‐stained particles in the nucleoli indicates higher transcriptional activity within the NORs [30]. Compared with D0, the nucleoli of iPSCs exhibited a marked increase in darkness, with a significantly higher abundance of black particles observed at D2 and D4. The increased density of silver particles in the nucleoli of D2 and D4 indicated enhanced transcriptional efficiency of active rDNA loci during the reprogramming process. Additionally, the number of active NORs also increased, with approximately five active NORs observed at D2 and D4, as opposed to only two prior to reprogramming (Figure 2E). Linear optical density values displayed an intuitive quantification of the density of silver‐stained particles within the nucleolus (Figure 2F). The optical density curve exhibited a valley‐shaped profile with bimodal edges at D0, and a platform‐shaped curve in iPSCs. At D2, the optical density curve also displayed a platform‐shaped curve with a slight depression in the middle, and by D4 it closely resembled the curve observed in iPSCs (Figure 2F).

To further investigate nucleolar changes, the nucleolar ultrastructure was examined using a transmission electron microscope (TEM), as alterations in rDNA transcription are typically accompanied by modifications in nucleolar structure. As shown in Figure 2G, at D0, a conventional somatic cell reticular nucleolus with distinct liquid phase boundaries was observed. However, by D2, the nucleolus transitioned to an intermediate state, whose structure was lacking clear liquid phase boundaries. By D4, the nucleolus with reappearing liquid phase boundaries, resembling the reticular nucleolus in iPSCs, was noted (Figure 2G and Figure S4D).

Our findings demonstrate significant changes in nucleolar morphology, structure and activity within the initial 2 days of iPSCs reprogramming. These changes, characterised by enlarged nucleoli, increased NORs activity, and the formation of a reprogramming intermediate nucleolar ultrastructure, are identified as an early event in the reprogramming process—nucleolar remodelling.

### 2.3 Oct4 Binds to rDNA Enhancer Region to Facilitate rDNA Transcription

**FIGURE 3:**
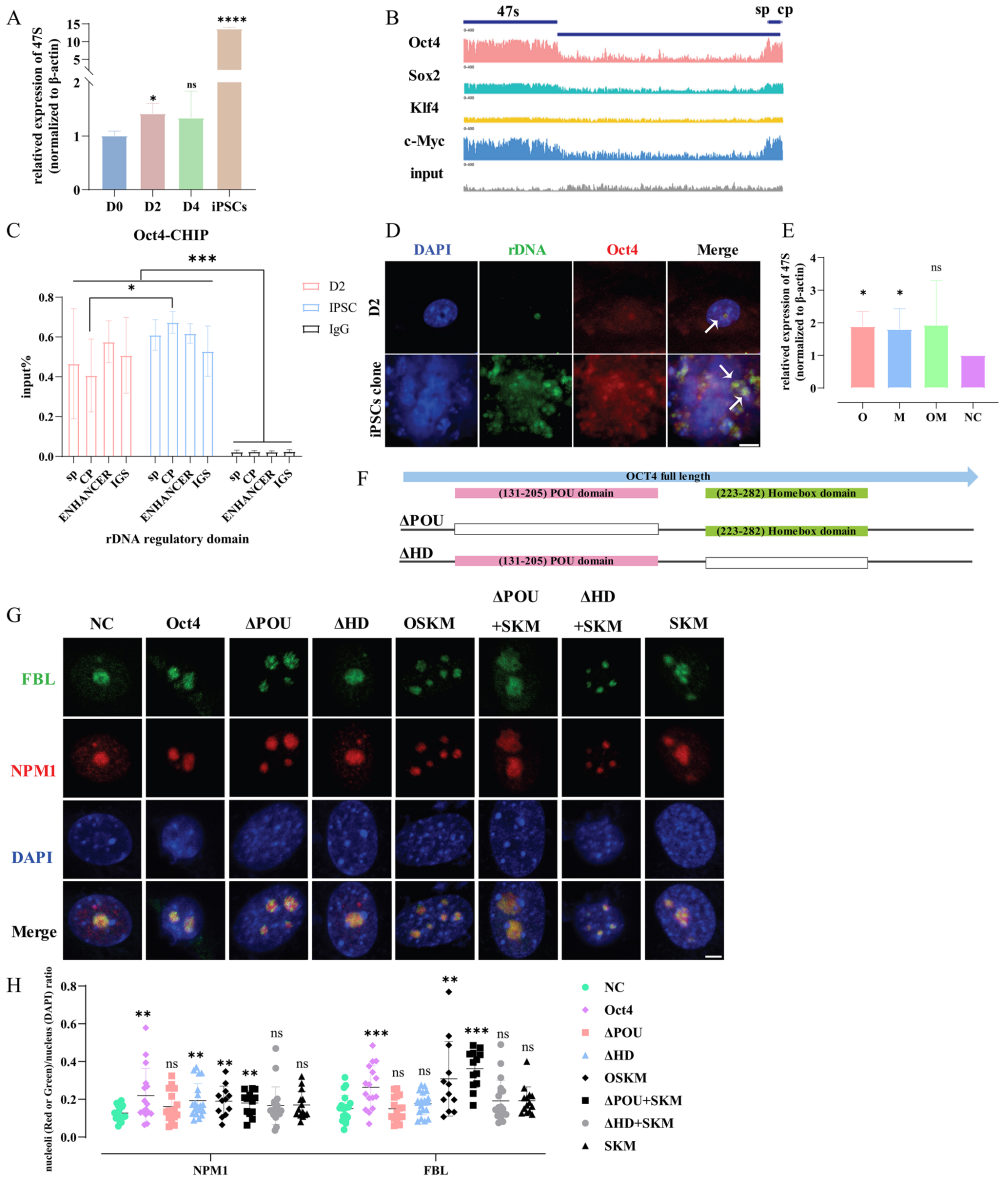
Oct4 binds to the rDNA enhancer region and promotes rDNA transcription. (A) RT‐qPCR analysis of 47S in the early stage of iPSCs reprogramming (D0, D2, D4) and iPSC, normalised to β‐actin mRNA levels. *p < 0.05, **p < 0.01, ***p < 0.001 (*n* = 3, Student's *t*‐test, mean ± SD). (B) ChIP‐seq profiles of OCT4, SOX2, KLF4 and c‐MYC at rDNA in D2 of iPSC reprogramming (GSE90895). Signal intensity is plotted on the *Y*‐axis. Intensity range is 0–400. (C) ChIP‐qPCR analysis of OCT4 binding to rDNA regulatory loci in D2 of iPSC reprogramming cells. *p < 0.05, **p < 0.01, ***p < 0.001 (*n* = 3, Student's *t*‐test, mean ± SD). (D) rDNA fish (green) and immunostaining of OCT4 (red) in D2 of iPSC reprogramming cells. The white arrow indicates the co‐localisation of rDNA and OCT4. Scale bar, 10 μm. (E) RT‐qPCR analysis of 47S in MEFs with Oct4 overexpression. *p < 0.05, **p < 0.01, ***p < 0.001 (*n* = 3, Student's *t*‐test, mean ± SD). (F) Diagram illustrating the model of Oct4 featuring domain‐specific mutations. (G) Immunostaining of NPM1 (red) and FBL (green) in the MEF with Oct4 overexpression or OCT4 with domain‐specific mutation. Scale bar, 10 μm. (H) Analysing the area ratio of five nucleolar protein (red or green) to DAPI (blue) in (G). Each dot represents nucleolus/nucleus ratio in one cell. *p < 0.05, **p < 0.01, ***p < 0.001, Student's *t*‐test, mean ± SD).

Chromatin in the nucleolus is mostly rDNA, which serves as the precursor for the production of rRNA transcripts. Given that rRNAs are typically excluded as background noise during RNA‐seq analysis, we evaluated the abundance of 47S, the initial transcript of rDNA, using RT‐qPCR. This evaluation confirmed an increased transcriptional activity of rDNA during iPSCs reprogramming within 48 h (Figure 3A). To investigate the underlying mechanisms of 47S rRNA elevation, we systematically evaluated two potential contributing factors: increased 47S rRNA half‐life and potential maturation defects. The degradation kinetics of 47S rRNA were quantitatively analysed at different stages of reprogramming to assess its turnover rate, which is unchanged (Figure S5A). To further address the concern that the observed increase in 47S levels might be caused by processing stalling, we measured the 18S content, which also showed an increasing trend during the early stages of reprogramming (Figure S5B). How is the rDNA transcriptional activity upregulated? We hypothesised that the four reprogramming factors (OCT4, SOX2, KLF4 and c‐MYC) target rDNA during the early stage of reprogramming.

To analyse mouse rDNA by ChIP‐seq, the obtained data were mapped to the mouse genome which contains a single copy of the mouse rDNA repeat. We examined the distribution of four factors on rDNA at D2 of iPSC reprogramming and observed the significant enrichment of OCT4 and c‐MYC on the two major regions of rDNA. These regions encompass the coding sequence, spanning from 0 to13.403 kb of the repeat, as well as regulatory regions, including spacer promoter (SP) and core promoter (CP). Notably, the peak values for CP and SP in OCT4 and c‐MYC groups reached approximately 180, whereas the input control displayed a value of only 20. SOX2 exhibited a comparable distribution pattern in this region, albeit with a somewhat diminished signal intensity, whereas KLF4 was markedly absent (Figure 3B). Previous research has indicated that c‐MYC plays a role in enhancing rDNA transcription activity [31]. To investigate whether OCT4 also accelerated rDNA transcription, we conducted ChIP‐qPCR analysis at the D2 stage of reprogramming. Specifically, we scrutinised the binding of Oct4 to various regions of the rDNA gene using region‐specific primer sets; of them, the ENHANCER region constitutes a segment of the SP region (Figure S5C). Our results indicate that Oct4 preferentially binds to the SP region at D2 reprogramming, with lesser affinity for the CP region (Figure 3C). Intriguingly, OCT4 binds to both the SP and CP regions of rDNA in fully reprogrammed iPSCs (Figure 3C). There is also an enrichment of OCT4 in the IGS region, as demonstrated by CHIP‐qPCR. We queried the corresponding IGS region enrichment in the ChIP‐seq data and found that the peak value for the OCT group in the IGS region is nearly 50, whereas the peak value for the input control is only 20. This finding suggests that OCT4 may play a regulatory role in the IGS region.

Additionally, the in vivo colocalization of OCT4 with a DNA probe specifically tagged to rDNA reinforces our findings (Figure 3D). Considering the binding site of OCT4 on rDNA, we propose that OCT4's role is primarily centred on regulating the enhancer region of rDNA, rather than directly modulating transcription initiation at the outset of iPSC reprogramming. Subsequent overexpression of *Oct4* in MEF demonstrated its ability to enhance rDNA transcription (Figures S5D and S5E; Figure 3E). Our observations suggest that the activation of rDNA transcription could be initiated by either OCT4 or c‐MYC. The co‐overexpression of OCT4 and c‐MYC demonstrated a rising trend, although the observed difference was not deemed statistically significant (Figure 3E). This occurrence may be linked to the possibility that rapid cellular proliferation without cell fate transformation attained contact inhibition, consequently influencing the transcriptional activity of rDNA [32].

OCT4 is composed of two essential domains—the POU domain and the homeobox domain (HD). To investigate how the OCT4 influences nucleolar activity, we deleted these two domains separately (Figure 3F). Our investigation revealed that although a defective POU domain in OCT4 still elicited nucleolar enlargement, a defective HD in OCT4 did not produce this effect (Figure 3G,H). Hence, it is conceivable that the HD of OCT4 plays a more crucial role in stimulating rDNA transcriptional activity and initiating nucleolar remodelling in comparison to the POU domain.

### 2.4 rDNA Transcription Is Crucial to the Nucleolar Remodelling

To further determine the necessity of activation of rDNA transcription in the context of nucleolar remodelling events, we inhibited rDNA transcription concurrently with the induction of reprogramming. Given the vast number of rDNA copies, knockdown approaches are infeasible; therefore, we consulted previous research to select a variety of inhibitors, actinomycin D (ActD), BMH‐21 and CX‐5461, to eliminate the off‐target effect. ActD accumulates within the polymerase molecules, disrupting the initiation of RNA synthesis. Notably, rRNA exhibits a significantly higher susceptibility to inhibition by ActD compared with mRNA, with a reported sensitivity up to 100‐fold greater [33]. BMH‐21 intercalates into GC‐rich regions of the rDNA to inhibit Pol I transcription [34]. CX‐5461 could inhibit the recruitment of Pol I to the rDNA and prevent the initiation of rDNA transcription by impeding the binding of the SL‐1 complex to the rDNA promoter or inhibiting the topoisomerase 2α (TOP2A) activity [35, 36, 37].

**FIGURE 4:**
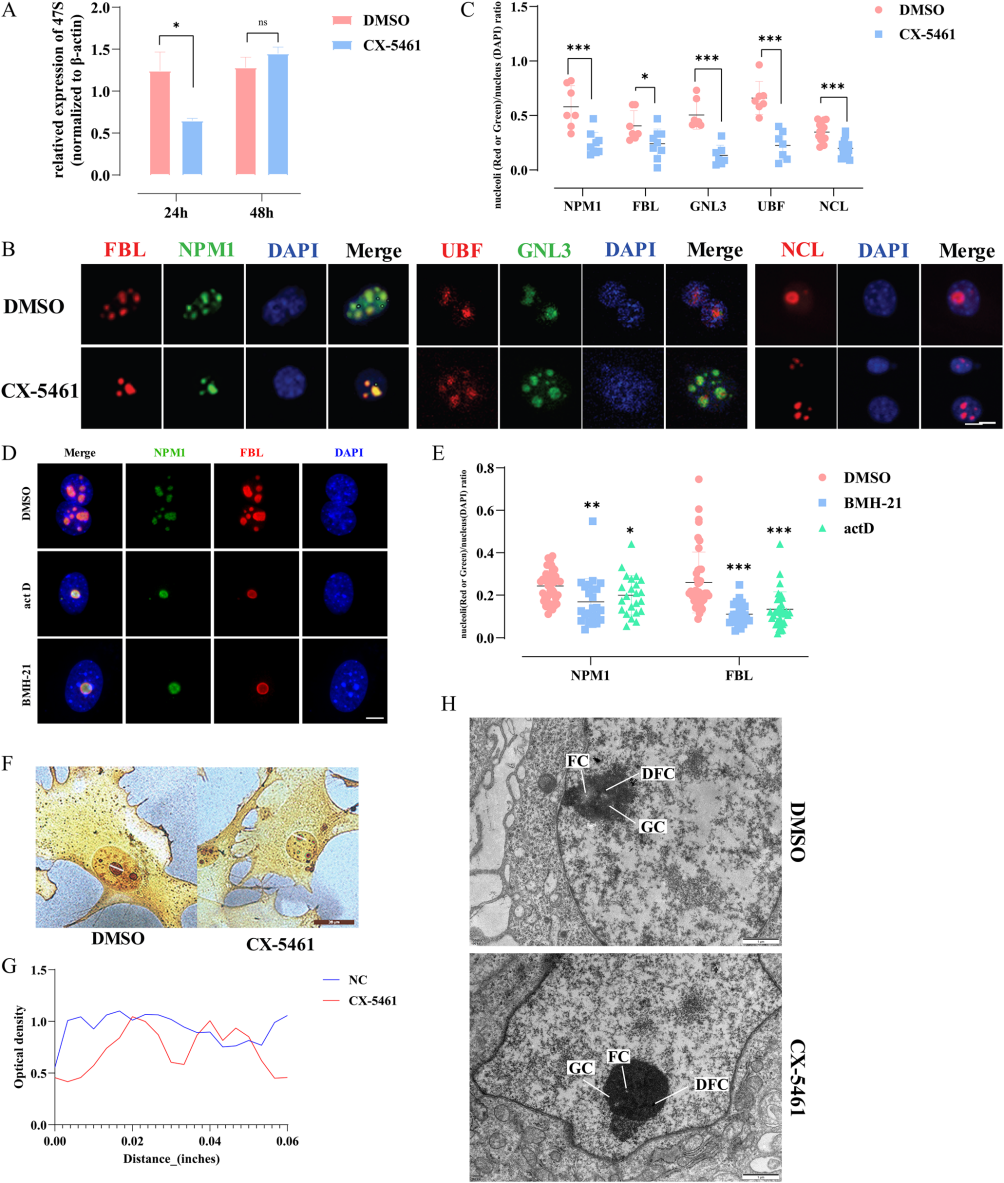
rDNA transcription was crucial to the nucleolar remodelling. (A) RT‐qPCR analysis of 47S in 4F2A MEFs (4TF) treated by DMSO (NC) or CX‐5461 (50 nM, 1 h) in reprogramming D0, normalised to β‐actin mRNA levels. The time points of sample collection were set at 24 and 48 h after dosing. *p < 0.05, **p < 0.01, ***p < 0.001, ****p < 0.0001 (*n* = 3, Student's *t*‐test, mean ± SD). (B) Immunofluorescence of five nucleolar proteins in the 4F2A MEFs treated with DMSO (the top row) or CX‐5461 (the bottom row) in reprogramming D0. Scale bar, 10 μm. (C) Analysis of area ratio of five nucleolar proteins to DAPI in (B), respectively. Each dot represents a nucleolar. *p < 0.05, **p < 0.01, ***p < 0.001 (*n* = 7–9, Student's *t*‐test, mean ± SD). (D) Immunostaining of NPM1 and FBL in the 4F2A MEFs treated with DMSO, BMH‐21 or ActD in reprogramming D0. Scale bar, 10 μm. (E) Analysis of area ratio of five nucleolar proteins to DAPI in D, respectively. Each dot represents a nucleolus. *p < 0.05, **p < 0.01, ***p < 0.001 (*n* = 7–9, Student's *t*‐test, mean ± SD). (F, G) Silver staining of NORs in the 4F2A MEFs treated by DMSO (left) or CX‐5461 (right) in reprogramming D0 (F), and analysis of silver stain particle density by optical density (G). Scale bars, 20 μm. (H) Nucleolar ultrastructure in the 4F2A MEFs treated by DMSO (left) or CX‐5461 (right) in reprogramming D0 was observed in reprogramming D2 by TEM. Scale bar, 1 μm.

CX‐5461 was used in a variety of concentrations. To avoid the effects of cell viability on iPSCs reprogramming efficiency, MEFs were exposed to a gradient of CX‐5461 concentrates ranging from 25 nM to 1 μM. (Figure S6A). Subsequent proliferation assays using CCK8 revealed that concentrations of 25 and 50 nM had negligible effects on cell viability compared with the blank and DMSO control groups. However, concentrations exceeding 50 nM led to a significant decrease in cell proliferation, evidenced by a reduction in absorbance values at 450 OD. To inhibit rDNA transcription effectively, MEFs were treated with 50 nM CX‐5461 and subsequently withdrawn after 1 h. RNA samples were collected for 47S detection at 24 and 48 h post‐CX‐5461 treatment. The RT‐qPCR results showed that 47S markedly decreased at 24 h but recovered by 48 h (Figure 4A). Given that CX‐5461 at a 50 nM concentration for 1 h resulted in effective and reversible rDNA transcriptional inhibition that was restored by D2, we further analysed the morphological changes at D2 in greater detail. Similar to D0, a nucleolar size of approximately 0.2 was observed at D2 of iPSC reprogramming, which failed to reach 0.5 as observed in the DMSO group (Figure 4B,C).

Transient treatment with ActD and BMH‐21 likewise exhibited the identical effect of inhibiting nucleolar enlargement (Figure 4D,E). Furthermore, there was no statistically significant difference in the level of nucleolar proteins between cells treated with CX‐5461 or DMSO, indicating that the decrease in relative nucleolar size was attributable to alterations in distribution rather than an alteration in protein abundance (Figure S6B). Likewise, the density of silver‐stained particles in NORs was significantly lower in the CX‐5461 group compared with the DMSO group. The NORs were shallower in the CX‐5461 group than in the DMSO group (Figure 4F). Furthermore, the sunken optical density curve in the CX‐5461 group appearedless full than in the DMSO group (Figure 4G).

Under a transmission microscope, the nucleolus in the CX‐5461 group appeared hyperchromatic and reticular, with distinct liquid phase boundaries. Conversely, the nucleoli in the DMSO group appeared as a loose and blurred liquid phase boundary structure. The nucleolus did not shift to reprogramming intermediate nucleolus at D2 of iPSC reprogramming with reduced transcriptional activity of rDNA by CX‐5461 treatment (Figure 4H and Figure S6C). The typical phenomenon of nucleolar remodelling observed in the rDNA inhibition group with inhibitor treatment was absent at D2. This suggests that transient and reversible inhibition of rDNA transcription impeded the nucleolar remodelling process, resulting in shrunken nucleoli, reduced activity of NORs, and a densified nucleolar ultrastructure.

### 2.5 Disturbing Nucleolar Remodelling Impairs Reprogramming Efficiency Only in the Early Stage

**FIGURE 5:**
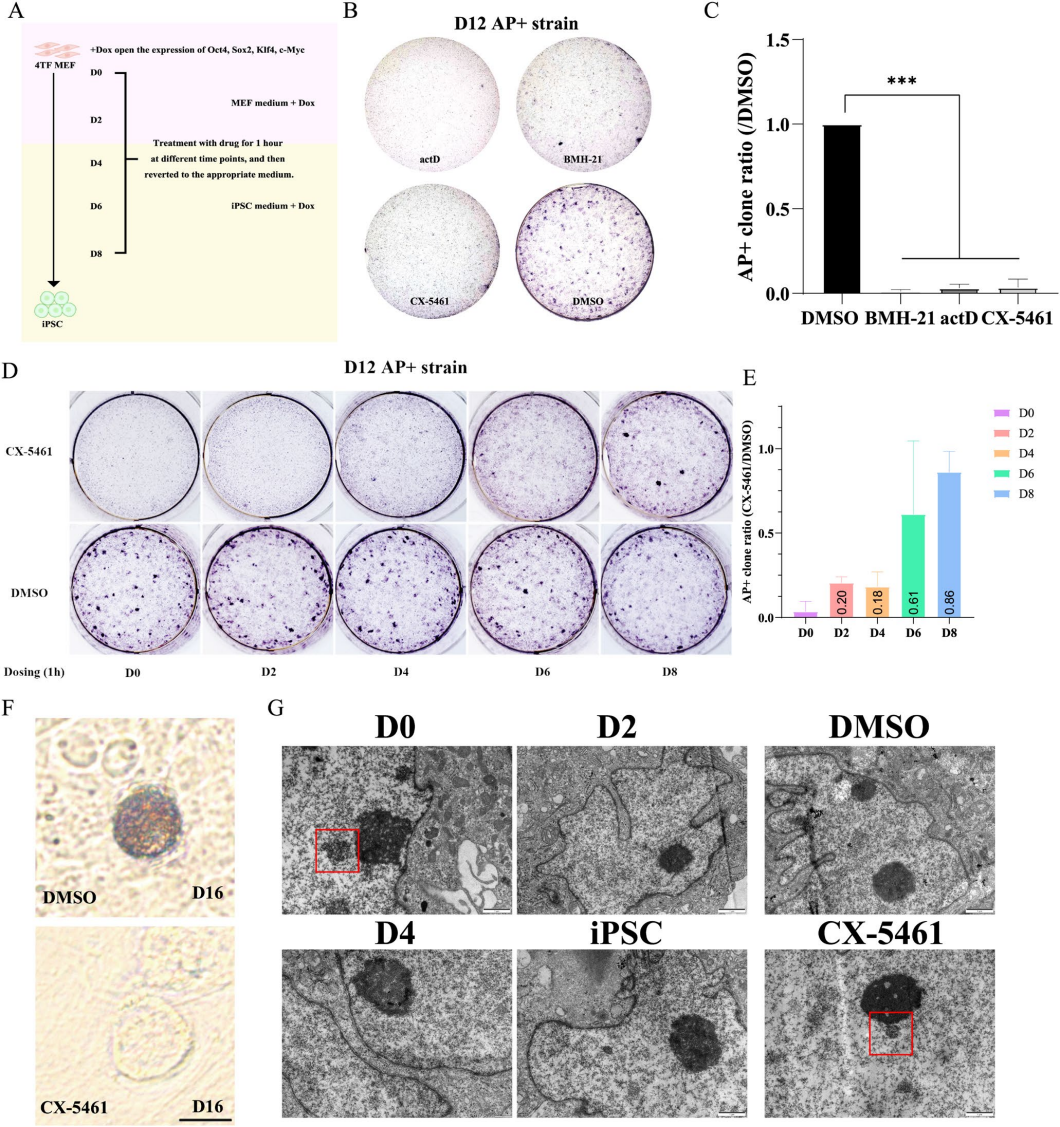
Disturbing nucleolar remodelling impaired reprogramming efficiency only in the early stage. (A) Schematic representation demonstrated 4F2A MEFs treated by inhibitors at different time points with the reprogramming process. (B) 4F2A MEFs following treatment with 50 nM CX‐5461, 80 ng/μL ActD, or 1 μM BMH‐21 for 1 h at reprogramming D0, the AP staining of 4F2A MEFs indicates their pluripotency at reprogramming D12. (C) The statistical graph indicates the ratio of AP^+^ clones in the CX‐5461, ActD, or BMH‐21 group to the NC group (*n* = 3). (D) 4F2A MEFs following treatment with DMSO or CX‐5461 (50 nM, 1 h) in reprogramming D0, the AP staining of 4F2A MEFs indicates their pluripotency at reprogramming D12. The statistical graph indicates the ratio of AP^+^ clones in the CX‐5461 group to the NC group. (F) 4F2A MEFs treated by DMSO or CX‐5461 (50 nM, 1 h) in reprogramming D0, the morphology of mature colons was formed and photographed in reprogramming D16. Scale bar, 50 μm. (G) TEM images reveal the nucleolus and its surrounding regions under different treatment conditions. The red circle indicates agglutinated heterochromatin. Scale bar, 1 μm.

Next, we investigated whether the nucleolar remodelling influences the eventual generation of iPSCs. MEFs were treated with 50 nM CX‐5461, 80 ng/μL ActD, or1 μM BMH‐21 at reprogramming D0 for 1 h (Figure 5A). All three inhibitors of rDNA transcription almost completely impaired iPSC lineage formation (Figure 5B). The elevated expression of alkaline phosphatase (AP) in stem cells compared with somatic cells establishes AP staining as a standard methodology for visualising successfully reprogrammed stem cell colonies during the late stages of cellular reprogramming. Upon distinction of the stem cell colonies via AP staining, utilising the imageJ software for quantification of the AP^+^ colonies, it was observed that the pharmacological treatment resulted in a marked reduction in the clone count (Figure 5C). In the DMSO control group, early clones were visualised by AP staining at D12 and mature clones were observed at D16 (Figure 5D,F), but no clones were found in the D0 treatment group. However, when the MEFs were treated by rDNA transcriptional inhibitor respectively at D0, D2, D4 and D6 during reprogramming, we observed several clones appeared in the D2 and D4 treatment groups (Figure 5D). Interestingly, there was relatively little difference in AP^+^ clone numbers between the D6 treatment and the DMSO group. Such results indicated that, as the CX‐5461 treatment was delayed, the difference in the AP^+^ clone numbers between the treatment and DMSO groups decreased, with the ratio approaching 1 at D8 (Figure 5E). As demonstrated in Figure 3, nucleolar remodelling was nearly completed by D4. In the D0 treatment group, rare clones with clear margins could be seen at D16, whereas in the DMSO group, mature clones with clear margins and bulged centres were observed at D16 (Figure 5F). Therefore, our results suggested that the inhibition of rDNA transcription notably hinders iPSCs reprogramming during nucleolar remodelling, with less effect on the establishment of iPSCs lines after nucleolar remodelling. Specifically, the inhibition of rDNA transcription in the initial stage of iPSC reprogramming impeded nucleolar remodelling, ultimately resulting in impaired iPSC generation. This highlights the significant influence of nucleolar remodelling on the early stages of iPSC reprogramming.

Eden Fussner et al. [38] reported that H3K9me3 was specifically enriched in heterochromatin by light microscopy/electron spectroscopic imaging (LM/ESI), and they demonstrated that compacted chromatin in MEFs converted to loosely packed chromatin in iPSCs for acquisition of the fully reprogrammed state. Here we discovered a similar result by TEM. We also revealed that perinucleolar heterochromatin (red frame) dynamics a temporal progression correlated with reprogramming stages (Figures 2G, 4H and 5G). At the initiation phase (D0), 41.9% of cells exhibited prominent perinucleolar heterochromatin aggregation, likely reflecting a transcriptionally repressed chromatin configuration. The significant reduction to 24.0% at D2 coincides with early nucleolar remodelling events, suggesting active disassembly of these heterochromatic structures during rDNA transcriptional activation. By D4, only 15.0% of cells retained residual perinucleolar heterochromatin and only 18.2% in iPSCs. Notably, CX‐5461‐mediated inhibition of rDNA transcription (43.8% positivity vs. 24.0% in DMSO controls) effectively preserved perinucleolar heterochromatin architecture, demonstrating that rDNA transcriptional activity is mechanistically required for chromatin state transition (Figure S7A). Significant variability exists in the gene composition of perinucleolar chromatin among various cell types [39]. Subsequently, we conducted an investigation to identify the specific genes that were released as a result of nucleolar remodelling.

### 2.6 Disturbing Nucleolar Remodelling Leads to a Disordered Chromatin Landscape

**FIGURE 6:**
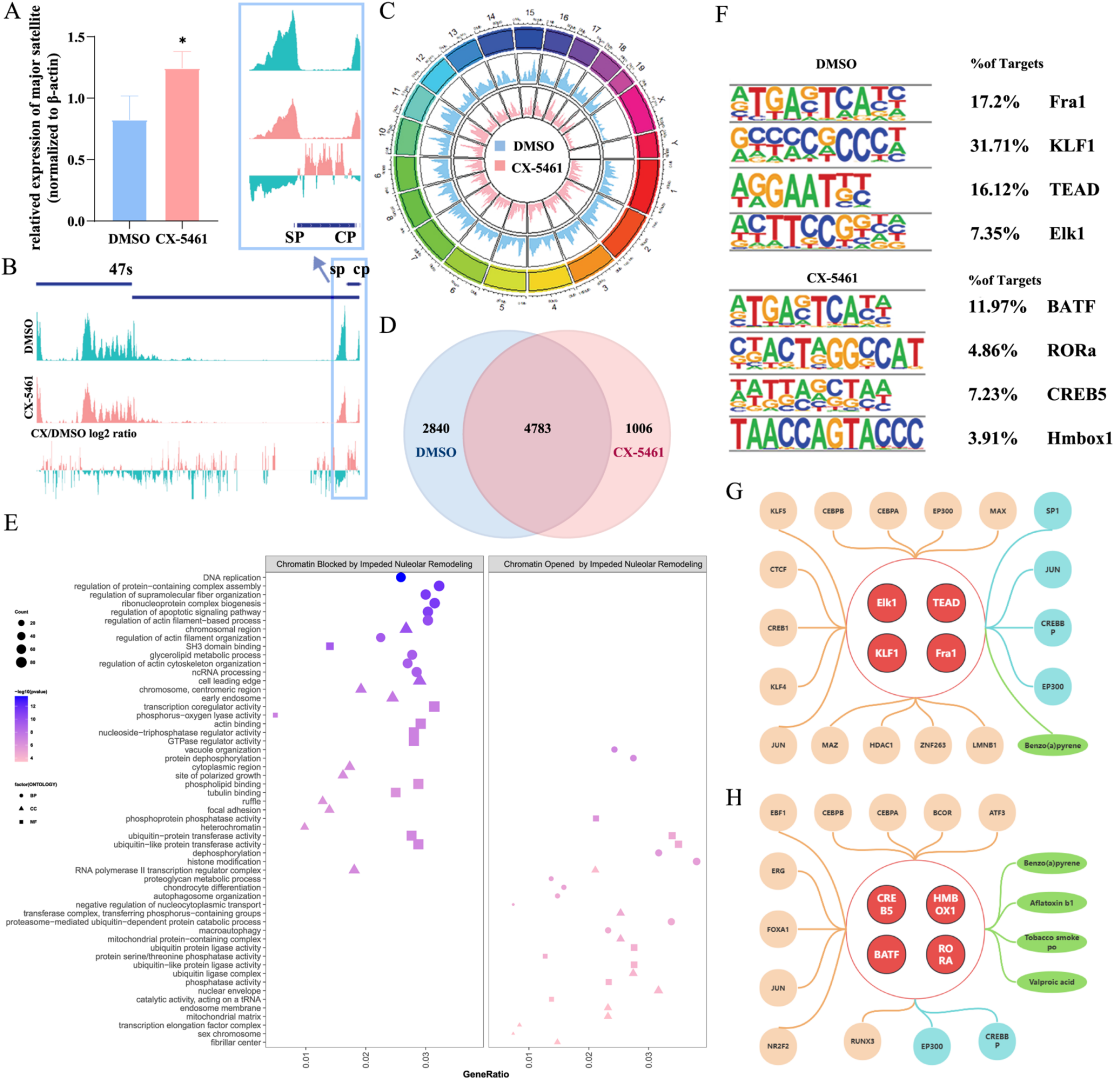
Disturbing nucleolar remodelling led to a disordered chromatin landscape. (A) RT‐qPCR analysis shows major satellite sequence in 4F2A MEFs (4TF) treated by DMSO (NC) or CX‐5461 (50 nM, 1 h), normalised to β‐actin mRNA levels in reprogramming D0. After inhibiting rDNA transcription, the expression level of the major satellite sequences is significantly increased. *p < 0.05, **p < 0.01, ***p < 0.001 (*n* = 3, Student's *t*‐test, mean ± SD). (B) The ATAC‐seq peaks reflect the degree of rDNA openness in reprogramming D2 after 4F2A MEFs (4TF) treated by DMSO (NC) or CX‐5461 (50 nM, 1 h) in reprogramming D0. The log2 ratio of the two groups (CX‐5461/DMSO) visually illustrates the changes in rDNA accessibility. Regions above the line correspond to areas with relatively higher rDNA accessibility following CX‐5461 treatment, whereas regions below the line represent areas with reduced rDNA accessibility after CX‐5461 treatment. The magnification is the promoter region. (C) Genome‐wide chromatin accessibility of DMSO (outer ring) and CX‐5461 (inner ring) group. The coloured ring represents the different chromosomes. (D) Venn diagram shows ATAC‐seq unique annotated peaks in DMSO and CX‐5461 group. DMSO and CX‐5461 represent the genes corresponding to the characteristic peaks identified in the respective groups from the ATAC‐seq analysis. ‘Up’ and ‘down’ refer to the genes that are upregulated or downregulated in the CX‐5461 group compared with DMSO. (E) GO enrichment analysis on the distinct genes identified with the DMSO and CX‐5461 groups. ‘Chromatin blocked by impeded nucleolar remodelling’ is enriched by lower degree of openness in the CX‐5461 group. Another is enriched by the genes that are enriched in the CX‐5461 group, where the chromatin is more open compared with the control group. (F) Motif enrichment analysis in the specific ATAC‐seq peaks upon DMSO or CX‐5461 treatment. (G) Regulatory network of transcription factors of unique ATAC‐seq peaks in the DMSO group. Red dots indicate transcription factors from Figure S6F. Orange dots represent their target genes. Blue dots indicate interacting proteins, and green dots show chemical molecules interacting with the red dot transcription factors. (H) Regulatory network of transcription factors from unique ATAC‐seq peaks in the CX‐5461 group. Red dots denote transcription factors from Figure S6F, orange dots are their target genes, blue dots are interacting proteins, and green dots highlight chemical molecules interacting with the red dot transcription factors.

Firstly, we attempt to elucidate the bridge between nucleolus and released heterochromatin during nucleolar remodelling. Our analysis indicated there were no significant differences in the levels of H3K9me3 between the CX‐5461 treated group and the DMSO group (Figure S7B). Furthermore, immunofluorescence staining did not reveal any notable variations in the colocalization of H3K9me3 (green) and nucleoli, as indicated by NPM1 (red), following treatment with CX‐5461 compared with the DMSO group (Figure S7C). The Pearson's *R* value was 0.3 in the DMSO group and was 0.26 in the CX‐5461 group (Figure S7D). A study has shown that the rDNA transcription could directly regulate the major satellite transcription through DNA–DNA 3D interaction, as shown by DNA‐FISH [40]. Given that heterochromatin formation involves not only proteins but also major satellite sequences, we examined the transcript levels of major satellite sequences, revealing a notable rise in the CX‐5461 treated group (Figure 6A).

To assess the chromatin landscape in detail for more rDNA‐interacting genes, an ATAC‐seq was conducted 2 days after iPSC reprogramming began, with or without CX‐5461 treatment. It was observed that chromatin accessibility of SP and CP of rDNA in the CX‐5461 group was reduced compared with the DMSO group (Figure 6B). Additionally, the chromatin openness of the promoter of five nucleolar proteins remained unaffected, consistent with previous findings that the change in nucleolar size was attributable to alterations in distribution rather than protein abundance (Figure S7E). The treatment of CX‐5461 resulted in a slight down‐regulation of global chromatin openness (Figure S7F). A ring graph depicting genome‐wide chromatin accessibility peaks under differential treatment conditions is presented. The outer ring displays chromosomal coordinates. Distinct spatial distribution patterns of chromatin accessibility were observed between the two experimental groups, as evidenced by differential peak loci in the pink (CX‐5461 treated) versus blue (DMSO control) chromatin accessibility tracks (Figure 6C). To systematically investigate the biological implications of these differential chromatin regions, we performed comprehensive functional annotation of the associated genomic loci. Analysis of unique peaks in the two groups revealed 2840 characteristic genes exclusive to the DMSO group and 1006 characteristic genes exclusive to the CX‐5461 group (Figure 6D).

GO analysis of the 2840 closed genes after CX‐5461 treatment indicated associations with DNA replication, and the cell component located mainly in the chromosomal region (Figure 6E, left). Conversely, the 1006 genes that opened after CX‐5461 treatment were mainly related to protein post‐translational modification (Figure 6E, right). The analysis revealed that the peaks observed exclusively in the DMSO group exhibited a preference for the Fra1, KLF1, TEAD and Elk1 motif, whereas the TF‐motif analysis of the peaks in the CX‐5461 group revealed an enrichment of BATF, RORa, CREB5 and Hmbox1 binding sites (Figure 6F). The target genes co‐regulated by Fra1, KLF1, TEAD and Elk1 included *HDAC1* and *Ctcf*, contributing to the epigenetic regulation of chromatin and 3D genome conformation [41]. The protein partners interacting with these DMSO motif factors were CREBBP and EP300, which are also involved in the epigenetic regulation of chromatin and are essential for tumorigenesis [42] (Figure 6G). The CX‐5461 treatment group (Figure 6H) exhibited distinct transcriptional regulatory patterns compared with the DMSO control (Figure 6G). Specifically, key transcription factors including BATF, RORa, CREB5 and Hmbox1 predominantly regulated the Atf3, Cebpa and other genes, which are functionally implicated in cell cycle regulation and cellular stress response pathways. Taken together, our analysis of annotated genes and enrichment peaks indicated a chromatin‐associated function, implying that the inhibitory impact of CX‐5461 on the initiation of iPSC reprogramming is linked to an aberrant and less permissive chromatin landscape.

### 2.7 Disturbing Nucleolar Remodelling Downregulates Chromosome Accessibility Regulation‐Related Genes

**FIGURE 7:**
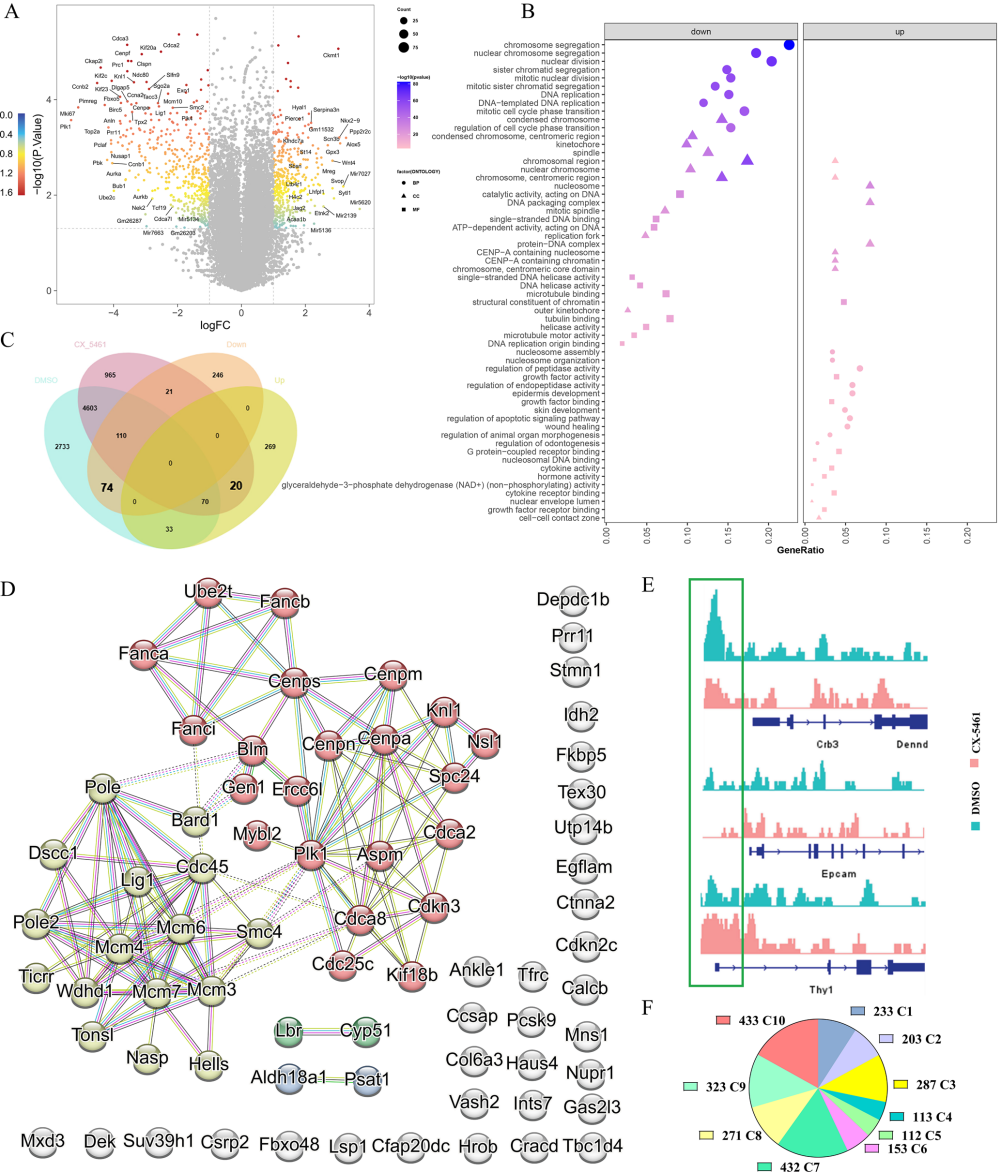
Disturbing nucleolar remodelling downregulated chromosome accessibility regulation‐related genes. (A) Volcanic map for differentially expressed genes between DMSO and CX‐5461 groups in D2 of iPSC reprogramming. Colourful plots represent significantly upregulated or downregulated genes. (B) GO enrichment analysis in the significantly upregulated and downregulated genes. (C) Venn diagram of ATAC‐seq unique peaks in DMSO and CX‐5461 group. DMSO and CX‐5461 represent the genes corresponding to the characteristic peaks identified in the respective groups from the ATAC‐seq analysis. ‘Up’ and ‘down’ refer to the genes that are upregulated or downregulated in the CX‐5461 group compared with DMSO. (D) Seventy‐four genes that are downregulated and undergo heterochromatinization after CX‐5461 treatment, compared with the DMSO control. The PPI network reveals their proteins are primarily involved in the cell cycle and chromatin remodelling. (E) The ATAC‐seq peaks reflect the degree of DNA openness at *Crb3*, *Epcam* and *Thy1* loci in reprogramming D2 after 4F2A MEFs (4TF) treated with DMSO (NC) or CX‐5461 (50 nM, 1 h) in reprogramming D0. (F) The pie chart shows the 2733 genes in each cluster of iPSC reprogramming. The cluster trends are seen in 1D.

Above findings prompted our further investigation into whether alterations in gene expression were a result of changes in chromatin accessibility. Subsequently, DEGs were filtered based on specific criteria to identify key functional genes (|log2FoldChange| ≥ 1 & *p* value < 0.05). Overall, 451 downregulated and 392 upregulated genes were screened (Figure 7A). These DEGs were mapped to terms of the GO database and found that downregulated genes were enriched in chromosomal segregation terms (Figure 7B, left), whereas MET‐related function appeared in the enriched terms of upregulated genes (Figure 7B, right). The downregulated genes identified in this study exhibited similar GO terms to ATAC‐seq peaks that were exclusively present in the DMSO group and were subsequently suppressed following CX‐5461 treatment. Through integration of ATAC‐seq and RNA‐seq data, our analysis focused on genes showing concordant changes in chromatin accessibility and gene expression in response to CX‐5461 treatment. We used a Venn diagram to illustrate the process of obtaining the intersection. DMSO and CX‐5461 represent the genes corresponding to the characteristic peaks identified in the respective groups from the ATAC‐seq analysis. ‘up’ and ‘down’ refer to the genes that are upregulated or downregulated in the CX‐5461 group compared with DMSO. Specifically, a total of 74 genes exhibited closed chromatin and downregulated gene expression, whereas 20 genes demonstrated open chromatin and upregulated gene expression (Figure 7C). These 74 downregulated genes were found to be involved in synergistic networks governing cell cycle regulation and chromatin accessibility (Figure 7D).

Among these genes, *Mybl2* and *Bard1* were critical for the chromatin landscape and crucial to MET during iPSC reprogramming [14, 43]. The *Crb3* and *Epcam* were markers of epiblast cells and upregulated for the MET event during iPSC reprogramming [44, 45]. However, chromatin accessibility of *Crb3* and *Epcam* was downregulated upon CX‐5461 treatment, and the chromatin changes of *Thy1*, the marker of mesenchymal cells, were opposite to the former (Figure 7E). Besides, there were 2733 genes whose chromatin was closed by CX‐5461 treatment, but gene expression did not significantly differ between the groups with or without CX‐5461 treatment (Figure 7C). The temporal expression patterns of these 2733 genes during iPSC reprogramming were visualised using a pie chart, in which trends of each cluster could be seen in Figure 1A (Figure 7F). Of these 2773 genes, cluster10 demonstrated the highest gene numbers, its genes exhibiting an upregulation during Days 2–4 of reprogramming. Our analysis revealed a notable increase in the expression levels of these genes during the intermediate and advanced stages of iPSC reprogramming. We observed a potential trend toward increased chromatin accessibility of these genes during the initial stages of iPSC reprogramming, suggesting a preparatory phase for their subsequent activation in later stages. From above, the boost of rDNA transcription induced by OCT4, which manifested by nucleolar remodelling, was found to be necessary for chromatin remodelling and preparation for the final establishment of iPSC.

In conclusion, our findings demonstrated that inhibition of rDNA transcription during the early stage of iPSC reprogramming blocked a cascade of reprogramming events, including nucleolar remodelling, chromatin remodelling and MET. These results detail the importance of activated rDNA transcription for the restoration of pluripotency in somatic cells.

## 3 Discussion

This research provides novel insights into the changes in rDNA transcription activity and nucleolar morphology during iPSC generation, characterising this phenomenon as nucleolar remodelling. This process acts as a doorman, facilitating alterations in chromatin structure, preceding MET and serving as an early indicator of somatic cell reprogramming.

It is noteworthy that both somatic cells and established stem cells exhibit reticular nucleoli with distinct levels of rDNA transcription activity, albeit at differing levels—lower in somatic cells and higher in stem cells. Interestingly, we found that the reprogramming process gives rise to an intermediate nucleolus characterised by hypertranscription of rDNA, termed a reprogramming intermediate nucleolus. As shown in our results, somatic reticular nucleolus transformed to a reprogramming intermediate nucleolus at D2 of iPSC reprogramming, and then to stem cell reticular nucleolus at D4. We found that gene expression patterns, nucleolar morphology, structure and function undergo drastic changes mainly in the D0–D2 stage. MET‐related genes mainly exhibit changes in D2–D4 and D4–D6. We also observed that the changes in cell morphology transforming from long fusiform shape to paving stone like also tended to occur around D4. Previously, it was believed that MET is the earliest event in iPSC reprogramming, during which MEF morphology changes from long spindle shaped to shorter forms and tends to aggregate. At the meantime, the mesenchymal genes turn off and the epithelial genes turn on [46]. Our results suggest that nucleolar remodelling occurs before MET. A cluster analysis of human reprogramming expression from GEO database supports our opinions, which showed that ribosome related genes are ascending to human induced pluripotent stem cells (h‐iPSCs) expression pattern within 48 h. However, the database did not indicate a similar occurrence of MET during this time period [47].

Furthermore, our results demonstrate that a temporary inhibition of rDNA transcription at the initiation of reprogramming results in the inability to generate iPSC. Recent research also has indicated that inducing LIN28A during reprogramming facilitates nucleolar segregation and enhances the efficiency of iPSC reprogramming [12]. However, the specific impact of nucleoli on reprogramming has not been further elucidated. During ESC differentiation, the formation of rDNA heterochromatin contributed to the development of peri‐nucleolar heterochromatin [48]. It provided a possible mechanism that the nucleolus regulated chromatin accessibility. However, due to the excessive copy number of rDNA, it is challenging to achieve overexpression, along with the absence of small molecule drugs that activate the nucleolus, which has hindered previous studies from conducting experimental validation of this hypothesis. Here, we directly inhibited nucleolar activation and identified downstream target genes, elucidating how reprogramming factors transiently induce a highly active remodelling state of nucleoli, releasing chromatin around the nucleoli, thereby enabling the expression of genes such as MET and subsequent events in reprogramming. Perinucleolar genes in ESCs mainly contain 2C‐related genes, whereas in 293T cells, they are more closely linked to neurodevelopment [6, 49]. Perinucleolar chromatin in somatic cells is implicated in factors important for differentiation and development [50]. Our results indicate that perinucleolar chromatin within reprogramming intermediate nucleoli is associated with chromatin remodelling and cell cycle regulation. Our results demonstrate a novel discovery in which the loosening of the nucleolus leads to the release of peri‐nucleolar heterochromatin. Furthermore, we have provided evidence to support the notion that inhibiting the relaxation of the nucleolus prevents the release of peri‐nucleolar heterochromatin. Through this elucidation, we have completed an important piece of the puzzle in understanding the nucleolar influence on heterochromatin. Nevertheless, advancements in technology are required to accurately visualise 3D rDNA–DNA interactions at a one‐to‐one level due to the constraints of visualising single‐copy genes with DNA‐FISH.

Besides, our research initially revealed that the hypertranscription of rDNA is crucial for the successful establishment of iPSC lines, which is dependent on the direct interaction between OCT4 and the rDNA enhancer region, thereby promoting transcription. Oct4, a pioneer transcription factor specific to stem cells, maintains the self‐renewal and undifferentiated state of ESCs by activating pluripotency‐related genes and repressing the expression of differentiation‐related genes [51]. Oct4 is characterised by two conserved domains, the HD and the POU domain, with studies indicating that the POU domain primarily mediates its functions [52]. However, the specific role of the HD domain has yet to be fully elucidated. Our research has revealed that the HD domain is involved in the regulation of nucleolar remodelling, suggesting a new link between nucleolar function and ESC differentiation. Inhibition of rDNA transcription, as well as Oct4 itself, can induce ESC differentiation, also highlighting the relation of OCT4 and rDNA transcription in maintaining the pluripotent state of stem cells [53, 54, 55]. In stem cells, the high expression of Oct4 may also serve the purpose of preserving rDNA transcriptional activity. Further investigation into its regulatory networks will be conducted.

Finally, mammalian development begins with a fertilised egg, in which the nucleolus manifested as a condensed solid structure known as a nucleolar precursor lacking rDNA transcription [56]. During the 2‐cell stage in mice, rDNA and ribosome biogenesis‐related proteins gradually enter into the nucleolar precursor, leading to the initiation of rDNA transcription [57]. During early embryonic development, blastomeres transition from totipotency to pluripotency, nucleoli transform from nucleolar precursors to stem cell reticular nucleoli in the inner cell mass (ICM) [58]. Line1 facilitates rRNA transcription, suppresses Dux expression and subsequently propels cells to exit the 2‐Cell state [55]. Pluripotent ESCs can be induced to transition into totipotent 2‐cell‐like cells (2CLCs) in vitro. Both in ESCs and during embryonic culture, inhibition of rRNA transcription disrupts nucleolar morphology and triggers the expression of 2C genes [58]. As the embryo develops, the stemness of the ICM diminishes, leading to differentiation into somatic cells, albeit with lower nucleolar activity compared with stem cells [59]. The process of somatic cell reprogramming can result in the transformation of a differentiated cell into an undifferentiated state. At present, two effective methods for somatic cell reprogramming are iPSC and SCNT. The differentiated somatic cell nucleus can be reprogrammed to a totipotent state in the cytoplasm of the oocyte, enabling it to develop into a complete organism [60]. iPSCs are generated by overexpressing four transcription factors in somatic cells, allowing them to regain pluripotency and differentiate into various tissues and organs. However, iPSCs do not possess the ability to develop into complete organisms [61]. Our previous study indicated that during SCNT, somatic cell nucleoli are initially remodelled into the nucleolar precursors and subsequently establish functional reticular nucleoli [8]. Here our findings shed light on the fundamental differences in nucleolar changes during two kinds of somatic cell reprogramming processes. The reticular nucleolus transforms into a nucleolar precursor in SCNT, but into a reprogramming intermediate nucleolus during the reprogramming of iPSCs. This coincides with a decrease in rDNA transcriptional activity in SCNT, whereas there is an increase in this process in iPSCs.

To sum up, in SCNT, rDNA transcription is suppressed, leading to nucleolar remodelling into nucleolar precursors and reacquisition of the totipotent state by differentiated cells. In iPSC reprogramming, the activation of rDNA transcription by reprogramming factors results in the remodelling of the nucleolus into a structure with blurred three liquid phase boundaries, facilitating the initiation of pluripotency in differentiated cells. Therefore, our hypothesis posits that the special loss of rDNA transcription induces the transformation of a nucleolar precursor, permitting the pluripotent stem cells to regain totipotency. Conversely, an upregulation of rDNA transcription triggers the nucleolar transformation from a reticulated nucleolus to a more active nucleolus, facilitating the acquisition of pluripotency by the cell. Our findings strongly support this hypothesis that the nucleolus was the doorman of cell fate, through which the cell may be on the path to corresponding destiny.

## 4 Materials and Method

### 4.1 Materials

Anti‐nucleostemin (ab70346), anti‐FBL (ab5821) and anti‐UBF (ab244287) were purchased from Abcam. Anti‐NPM1 (FC‐61991) was purchased from ThermoFisher Scientific. Anti‐α‐tubulin (9099S) was purchased from Cell Signalling Technology (CST). ProLong Gold Antifade Mountant with DAPI (P36931) and Alexa Fluor conjugated antibodies (A10042, A11004, A32790, A32723) were purchased from Invitrogen. CX‐5461 (HY‐13323) was purchased from MCE.

### 4.2 Mouse Experiments

C57BL/6 and DBA/2 mice at 6–8 weeks old were purchased from Vital River Laboratories (Beijing, China); 4F2A mice (expressing the dox‐inducible polycistronic 4F2A cassette consisting of four mouse reprogramming genes Oct4, Sox2, Klf4 and c‐Myc from the Col1a1 locus) were purchased from Jackson Laboratory (Bar Harbour, America). Primary 4F2A MEFs were derived from E13.5 4F2A embryos obtained by inbreeding of 4F2A 8‐week‐old mice, passed to generation 1, termed P1 4TF for reprogramming. Primary MEFs were obtained by crossing DBA/2 male mice with C57BL/6 female mice. Passed to P3 and treated with mitomycin to derive feeder cells. All animal procedures were approved by the Code of Practice Harbin Medical University Ethics Committees.

### 4.3 Cell Culture

MEFs, 4TF MEFs were cultured in DMEM/high glucose (Gibco, 12100046) supplemented with 10% FBS (Biological Industries, 04‐001‐1ACS), 1% penicillin–streptomycin (Gibco, 15140122) and 1% L‐glutamine (Gibco, 25030081). Mouse iPSCs were maintained in Knockout DMEM (Gibco, 10829018) supplemented with 20% KOSR (Gibco, 10828028), 1% penicillin–streptomycin (Gibco, 10378016), 1% L‐glutamine (Gibco, 25030081), 1% MEM NEAA (Gibco, 11140050), 1% sodium pyruvate (Gibco, 11360070), 0.1% 2‐mercaptoethanol (Gibco, 21985023) and 1000 U/mL LIF (Peprotech, 250‐02). To measure RNA stability, transcription was blocked by adding ActD to the medium at a concentration of 2.5 μg/mL. Cells were counted and harvested at 0, 1, 2 and 4 h after the addition of ActD.

### 4.4 iPSCs Generation

4TF MEF could be TetOn the expression of Oct4, Sox2, Klf4 and c‐Myc by doxycycline. 2–4 × 10^4^ 4TF MEF cells were seeded in a plate containing feeder cells with 0.1% doxycycline to medium and it is denoted as Day 0. The medium was changed to iPSCs medium supplemented with 0.1% doxycycline at Days 3–4. The number of AP^+^ colonies was counted at Day 12.

### 4.5 Total RNA Extraction and RT‐qPCR

Cellular total RNA was extracted according to the FastPure Cell/Tissue Total RNA Isolation Kit V2 protocol (RC112‐01, Vazyme). Extracted RNA was dissolved in deionised water and then reverse transcribed by using a HiScript II Q RT SuperMix for qPCR kit (R222‐01, Vazyme) according to the manufacturer's instructions. Real‐time PCR was performed by using Taq Pro Universal SYBR qPCR Master Mix (Q712‐02, Vazyme) according to the manufacturer's instructions and analysed on the CFX96 Real‐time System (Bio‐Rad). The primers used for RT‐qPCR were in the Table S1.

### 4.6 CHIP‐qPCR

ChIP assays were conducted using the SimpleChIP Enzymatic Chromatin IP Kit (Magnetic Beads) (Cell signalling technology #9003s) according to the manufacturer's instructions. Cells were cross‐linked with 1% formaldehyde (Sigma) and subsequently quenched with 0.125 M of glycine. Cells were collected with ChIP buffer and sonicated to obtain cross‐linked chromatin. The cross‐linked chromatin was incubated with antibodies overnight at 4°C, and then the protein G beads were added for further incubation. De‐crosslinked DNA was purified using a purification kit. The primers used for CHIP‐qPCR were in the Table S1.

### 4.7 Western Blot

After adjusting the protein concentration of samples to the same value, samples were electrophoresed at 80 V and transferred to a PVDF membrane at 300 mA. The membranes were blocked in 5% milk for 1 h at room temperature, and then incubated with primary antibody overnight. Secondary antibodies were incubated for 1 h at room temperature after washing the membranes three times in PBST.

### 4.8 RNA Library Construction and Sequencing

For iPSC reprogramming data, RNA samples were sent to BGI Corporation (Shanghai, China) by using a BGISEQ‐500 sequencer. For data of CX‐5461 and DMSO treatment, the total RNA was extracted according to the instruction manual of the TRIzol Reagent (Life technologies, California, USA). RNA concentration and purity were measured using NanoDrop 2000 (ThermoFisher Scientific, Wilmington, DE). RNA integrity was assessed using the RNA Nano 6000 Assay Kit of the Agilent Bioanalyzer 2100 system (Agilent Technologies, CA, USA). Next, mRNA was purified from total RNA using poly‐T oligo‐attached magnetic beads. A total amount of 1 μg RNA per sample was used as input material for the RNA sample preparations. Sequencing libraries were generated using Hieff NGS Ultima Dual‐mode mRNA Library Prep Kit for Illumina (Yeasen Biotechnology (Shanghai) Co. Ltd.) following the manufacturer's recommendations and index codes were added to attribute sequences to each sample. In details, first strand cDNA was synthesised and second strand cDNA synthesis was subsequently performed. Remaining overhangs were converted into blunt ends via exonuclease/polymerase activities. After adenylation of 3′ ends of DNA fragments, NEBNext Adaptor with hairpin loop structure were ligated to prepare for hybridization. The library fragments were purified with AMPure XP system (Beckman Coulter, Beverly, USA). Then 3‐μL USER Enzyme (NEB, USA) was used with size‐selected, adaptor‐ligated cDNA at 37°C for 15 min followed by 5 min at 95°C before PCR. Then PCR was performed with Phusion High‐Fidelity DNA polymerase, Universal PCR primers and index (X) primer. At last, PCR products were purified (AMPure XP system) and library quality was assessed on the Agilent Bioanalyzer 2100 system. The libraries were sequenced on Illumina NovaSeq 6000 platform to generate 150 bp paired‐end reads, according to the manufacturer's instructions. The raw reads were further processed with a bioinformatic pipeline tool, BMKCloud (www.biocloud.net) online platform. Raw data (raw reads) of fastq format were firstly processed through in‐house perl scripts. In this step, clean data (clean reads) were obtained by removing reads containing adapter, reads containing poly‐N and low‐quality reads from raw data. At the same time, Q20, Q30, GC content and sequence duplication level of the clean data were calculated. All the downstream analyses were based on clean data with high quality.

### 4.9 RNA‐Seq Data Bioinformatic Analysis

Trimmomatic was used in paired‐end mode to trim the adaptor and low‐quality sequence. Reads that aligned to the mm10 reference genome by hisat2 were counted by FeatureCounts. Counts were converted to tpm and were analysed for searching differential genes using the limma package, and genes with an adjusted *p* value < 0.05 and log2 fold change ≥ 1 were assigned as differentially expressed. clusterProfiler was used for GO and KEGG analysis [62]. Temporal clustering was performed using the R package mfuzz, with the number of clusters set to 10. PCA was performed on the transposed expression data using the default parameters of the prcomp package in R. The scatter plots and bar charts were visualised using the ggplot2 package, whereas the heatmap was generated using the pheatmap package. To facilitate verification by non‐bioinformatic readers, we developed an R package, Grouphmap, for differential analysis across multiple groups and for generating heatmaps of extracting multi‐group GO results. The source code and a comprehensive vignette are freely available at https://CRAN.R‐project.org/package=Grouphmap.

### 4.10 CHIP‐Seq Data Bioinformatic Analysis

Trimmomatic was used in paired‐end mode to trim the adaptor and low‐quality sequence. Reads that aligned to the mm10 reference genome, which was added the one 45 kb rDNA repeat as an extra chromosome, by bowtie2 (version 2.3.5.1). Peaks were identified using MACS2 and visualised by IGV. The peak threshold is set to 0–400.

### 4.11 ATAC Library Construction and Sequencing

ATAC‐seq was performed by Hyperactive ATAC‐seq Library Prep Kit for Illumina (TD711, Vazyme). A total of 50,000 cells were washed twice with 50 μL of TW buffer and resuspended in 50 μL lysis buffer (RS buffer, 11% NP40, 10% Tween, 1% digitonin). The suspension was then centrifuged for 10 min at 500 g at 4°C, followed by adding 50 μL transposition reaction mix (TW buffer, 10% Tween, 1% digitonin, ddH2O, 5*TTBL, TTE mix V50) and then incubated at 37°C for 30 min. 5‐μL stop buffer was added to stop the reaction. DNA was isolated by ATAC DNA Extract Beads. ATAC library was subjected to 17 cycles of amplification with TruePrep Index Kit V2 for Illumina (TD202, Vazyme). ATAC library was purified by ATAC DNA Clean Beads and measured using Qubit 4 fluorometer. Library integrity was checked by gel electrophoresis. Finally, the ATAC libraries were sequenced on Illumina NovaSeq 6000 platform to generate 150 bp paired‐end reads at BerryGenomics Corporation.

### 4.12 ATAC‐Seq Data Bioinformatic Analysis

Cutadapt was used in paired‐end mode to trim the adaptor sequence and separate sequences. Reads that aligned to the mm10 reference genome, which added the one 45 kb rDNA repeat as an extra chromosome, were processed by bowtie2 (version 2.3.5.1). ATAC‐seq peaks were identified using MACS2. Analysed by CHIPseeker and clusterProfiler in Rstudio [62, 63]. The coloured circular rings were visualised using the circlize package in R. The Venn diagram is generated from jvenn (http://www.bioinformatics.com.cn/static/others/jvenn/example.html) [64]. Regulatory networks were retrieved from the Gendoma web server (https://ai.citexs.com). The PPI network is generated from STRING 11.5 (https://cn.string‐db.org).

### 4.13 Plasmid Constructs

The pMXs‐Oct3/4 was acquired from addgene (Plasmid #13366). To construct four ΔpMXs‐Oct3/4, recombinant fragments (P4–7) were PCR‐amplified from pMXs‐Oct3/4 using the primers in Table S1. pMXs‐Oct3/4 was digested with NcoI‐HF (R3193V, Biolabs) and NsiI‐HF (R3127V, Biolabs), and the plasmid skeleton (P) was purified by agarose gel electrophoresis.

The pMXs‐Oct3/4‐ΔPOU is generated from P, P4 (F2/3/4+R3), and P5 (F3+R1/2/3/4) by homologous recombination. The pMXs‐Oct3/4‐ΔHD is generated from P, P6 (F2/3/4+R4), and P7 (F4+R1/2/3/4) by homologous recombination. The homologous recombination was performed by ClonExpress Ultra One Step Cloning Kit (C115, Vazyme) according to the manufacturer's instructions. Transformation was performed by DH5α (CB101, Tiangen).

### 4.14 DNA FISH

Probe of rDNA were synthesised according to the instructions of FISH Tag DNA Multicolor Kit (F32951, Invitrogen). For DNA FISH, cells were fixed with 4% paraformaldehyde and stored at 4°C, then rinsed with PBS (Solarbio, P1010). The cells were blocked in 0.1% Triton X‐100 (Solarbio, T8200) in PBS (PBST) containing 2% BSA at room temperature for 30 min. Then cells were incubated with 100 μg/mL RNase A in 2× SSC at 37°C for 45 min and 20 μg/mL Protease K for 3–6 min. After washing three times in 2× SSCT (2× SSC+ 0.1% Tween‐20), the samples were immersed in hybridisation buffer (70% formamide diluted in 2× SSC) for 30 min at 37°C then transferred to 85°C for 10 min. The probe mixed into hybridisation buffer, incubated at 72°C for 5 min and dropped on samples before hybridisation overnight. The cell samples above were washed in PBS and then incubated with primary antibody for the co‐immunostaining.

### 4.15 Single‐Cell RNA‐Seq Data Analysis

For the analysis of gene expression trends during the iPSC reprogramming, we used the public single‐cell data available under GSE242424. We directly used the processed data, metadata and cell state annotation deposited by the authors of GSE242424. UAMP was used for dimensionality reduction analysis.

### 4.16 Alkaline Phosphatase Staining

AP staining was performed by using a BCIP/NBT Alkaline Phosphatase Color Development Kit (Beyotime, C3206) according to the manufacturer's instructions. In brief, cells were washed three times with DPBS, then incubated with BCIP/NBT staining solution at room temperature in darkness until the cells got dyed. Washed cells were maintained in DPBS. AP^+^ clones were observed and imaged by light microscopy and analysed by ImageJ.

### 4.17 Immunofluorescent Staining

Cells were fixed with 4% paraformaldehyde and stored at 4°C, then rinsed with PBS (Solarbio, P1010). Then the cells were blocked in 0.1% Triton X‐100 (Solarbio, T8200) in PBS (PBST) containing 2% BSA at room temperature for 30 min, and incubated with specific primary antibodies overnight at 4°C. After that, the cells were rinsed three times at room temperature for 5 min per time, incubated with corresponding secondary antibodies conjugated with Alexa Fluor 568 or Alexa Fluor 488 for 1 h at room temperature in the dark. Finally, each sample was rinsed and placed on a glass slide with a drop of ProLong Gold Antifade Mountant with DAPI (Invitrogen, P36931) and compressed with a cover‐slip for imaging. All images were captured by a Nikon C2 confocal microscope and analysed by ImageJ. For area ratio, first, we performed channel splitting on the original immunofluorescence images. Then, processing the blue channel to identify the nuclei, and converting the segmented nuclei into regions of interest (ROIs). Finally, for each channel, we adjusted the appropriate threshold using ImageJ's auto‐segmentation function (CTRL + T) to delineate the region covering the highest‐intensity fluorescent foci, which were consistently smaller than DAPI, and predominantly localised within DAPI‐demarcated luminal compartments. Selective minor manual upward adjustment of the threshold was implemented in rare instances where the nucleolar and nucleoplasmic fluorescence intensity was not big enough, as the Otsu‐based auto‐thresholding occasionally under‐segmented these low‐contrast regions. This conservative refinement specifically targeted subtle signal gradients at nucleolar boundaries (representative examples of this threshold adjustment process was provided in Figure S8). Too often, the auto‐generated selected region showed strong concordance with visually identified high‐intensity zones, after which fluorescent areas in each ROI were quantified. Note that in the ‘set measurement’ options, ‘limit to threshold’ should be checked. Data used to calculate the nucleolar area ratio were provided in Table S3.

### 4.18 EU Staining

Silver staining was performed with a BeyoClick EU‐488 RNA Kit (Beyotime, R0301S) according to the manufacturer's instructions. In brief, incubating the cells with 1 mM EU for 2 h, fix the cells by adding 1 mL of fixation solution and incubating at room temperature for 15 min. Following fixation, remove the fixation solution and wash the cells three times with 1 mL of wash buffer per well, with each wash lasting 3–5 min. After washing, remove the wash buffer and permeabilize the cells by adding 1 mL of permeabilization solution, incubating at room temperature for 10–15 min. Remove the permeabilization solution and wash the cells 1–2 times with 1 mL of wash buffer per well, each wash lasting 3–5 min. Prepare the Click additive solution according to the manufacturer's instructions, and then mix the Click reaction mixture fresh. Incubate the cells with the reaction mixture at room temperature, protected from light, for 30 min. Finally, after the reaction, washed the cells with wash buffer and perform immunofluorescence or imaging under a fluorescence microscope and analysed by ImageJ. For the statistics of intracellular nascent RNA, we separated nucleoli or single cells after adjusting the threshold recognition (image‐adjust‐threshold, process‐binary‐fill holes, process‐binary‐watershed), and used the ROI function to calculate the fluorescence intensity of single nucleoli or cells.

### 4.19 Silver Staining

Silver staining was performed with an Argyrophilic Nucleolar Organizer Region (AgNOR) Associated Proteins Stain Kit (Solarbio, G2015) according to the manufacturer's instructions. In brief, cell climbing sheets were fixed with precooled 4% PFA for 30 min, then rinsed with distilled water for 10 min. The sheets were incubated with AgNOR Stain Solution at room temperature for 40 min, until they turned brown. After rinsing with distilled water for 2 min, the sheets were re‐stained with methyl green solution for 1–3 min, rinsed and dried in air. Finally, the sheets were sealed with Antifade Mounting Medium (Beyotime, P0126‐25 mL) for imaging and analysed by ImageJ. We converted the image to grayscale and measured the radial grayscale value distribution along the diameter of the silver‐stained centre.

### 4.20 Transmission Electron Microscopy

Cells were collected into 1.5‐mL tubes, then fixed with 2.5% glutaraldehyde overnight at 4°C. Next, the samples were washed 3–5 times in PBS and transferred to agaropectin, then cut into blocks. The agaropectin blocks were transferred into epoxy resin with a specific mould at 85°C. The epoxy resin blocks were cut into 0.66‐μm thin slices using an ultramicrotome, then the slices were transferred to glass slides and stained with toluidine blue for evaluation. Then, ultra‐thin 99‐nm sections of the selected epoxy resin samples were rapidly cut and transferred to copper mesh, stained with uranyl acetate (2 g in 100 mL 50% ethyl alcohol, stain 15–20 min at 35°C) and lead citrate (Reynolds' method, stain 15–20 min). Samples were imaged with an electron microscope for the ultrastructure of the nucleoli.

### 4.21 Statistical Analysis

ATAC‐seq and RNA‐seq were performed twice. All statistical charts were performed in GraphPad Prism 8. The data were analysed with Student's *t*‐test and presented as the mean ± SD. The differences were considered statistically significant at the *p* < 0.05 level (*p < 0.05, **p < 0.01, ***p < 0.001, ns, not significant).

## Author Contributions

**Yuchen Sun:** conception and design, collection and assembly of data, data analysis and interpretation, manuscript writing. **Xinglin Hu:** collecting the reprogramming cell samples of RNA‐seq for Figures 1 and 2, assist in discussion. **Xingwei Huang:** assist in discussion and experimental technical support. **Wenyao Zhou** and **Shubing Lan:** vector construction. **Hui Zhang:** financial support. **Guangming Wu** and **Lei Lei:** conception and design, financial support, administrative support, final approval of manuscript. All authors read and approved the final manuscript.

## Ethics Statement

The authors have nothing to report.

## Consent

The authors have nothing to report.

## Conflicts of Interest

The authors declare no conflicts of interest.

## Supporting information


**Figure S1.** Activation of nucleolar function‐related genes was mainly in the first 2 days of iPSC reprogramming. (A) Unsupervised clustering of mRNA expression in five iPSCs reprogramming stages analysed by RNA‐seq. (B) Principal component analysis of mRNA expression. The same colour represents the same iPSCs reprogramming stage in replicates. (C) Differential gene expression analysis of RNA‐seq data in each reprogramming period. The differential genes among D2 versus D0, D4 versus D2, D6 versus D4 and D8 versus D6 were displayed from left to right. (D) Volcanic map shows differentially expressed genes between D0 and D2. As red plots, significantly upregulated genes. As blue plots, significantly upregulated genes. Ribosome biogenesis related genes are extra noted. (E) The stack bar chart shows the percentage of rDNA transcription related genes (GO#0042790) in each cluster. The most distributed in C10 (red), and followed by clusters 2 and 5 (pink). (F) TPM of *DDX21*, *NCL*, *Nop16*, *Polr1a*, *Polr1e*, *Wdr36* and *Pwp1* in iPSC reprogramming is presented by line chart. (G) The pie chart shows the number of genes in each cluster in Figure 1A. (H) UMAP of single‐cell RNA‐seq labelled by characteristics of cells at different stage of human iPSC reprogramming.

**Figure S2.** Nucleolus‐related function active in the early stage of iPSCs reprogramming induced by OSKM. (A) Temporal clustering of RNA‐seq date of cells in OSKM iPSCs reprogramming (GSE137001). Fuzzy c‐means clustering identified seven distinct temporal patterns of gene expression in iPSCs reprogramming. The *X*‐axis represents four points in time for iPSCs reprogramming (D0, D2, D4, D6, D8), whereas the *Y*‐axis represents log2‐transformed, normalised intensity ratios in each stage. (B, C) GO and KEGG enrichment analysis of cluster 1. (D, E) GO and KEGG enrichment analysis of cluster 3.

**Figure S3.** Nucleolus‐related function active in the early stage of iPSCs reprogramming induced by SKM. (A) Temporal clustering of RNA‐seq date of cells in SKM iPSCs reprogramming (GSE137001). Fuzzy c‐means clustering identified eight distinct temporal patterns of gene expression in iPSCs reprogramming. The *X*‐axis represents four points in time for iPSCs reprogramming (D0, D2, D4, D6, D8), whereas the *Y*‐axis represents log2‐transformed, normalised intensity ratios in each stage. (B, C) GO and KEGG enrichment analysis of cluster 4. (D, E) GO and KEGG enrichment analysis of cluster 5.

**Figure S4.** 47S rRNA accumulation originates from enhanced rDNA transcriptional activation within nucleoli. (A) Analysing the immunofluorescence intensity of five nucleolar protein. *p < 0.05, **p < 0.01, ***p < 0.001, Student’s *t*‐test, mean ± SD. (B, C) 5‐EU staining in the early stage of iPSCs reprogramming (D2, D4), D0 is negative control and iPSCs is positive control. Scale bar, 200 px. The (C) diagram is a statistic of the fluorescence intensity in each cell in the D diagram. (D) The nucleolar region in Figure 2G were magnified and overexposed.

**Figure S5.** rDNA transcriptional activation and OCT4 can binds to rDNA. (A) At the stages of D0, D2, D4 during reprogramming and the iPSC stage respectively, samples with the same cell quantity were collected at 0, 1, 2 and 4 h after the addition of the actinomycin D (ActD). The content of 47S was detected by qPCR. The expression level at 0 h was set as 1, and the *Y*‐axis represents the content of 47S at each time point relative to that at 0 h. (B) RT‐qPCR analysis of 18S in the early stage of iPSCs reprogramming (D0, D2, D4) and iPSC, normalised to β‐actin mRNA levels. *p < 0.05, **p < 0.01, ***p < 0.001 (*n* = 3, Student’s *t*‐test, mean ± SD). (C) Diagram illustrating the primer of regulatory domain of rDNA. (D) RT‐qPCR analysis of Oct4 mRNA in MEF overexpressed Oct4. *p < 0.05, **p < 0.01, ***p < 0.001 (*n* = 3, Student’s *t*‐test, mean ± SD). (E) Detection of Oct4 expression through western blotting.

**Figure S6.** rDNA transcription was crucial to the nucleolar remodelling. (A) Cell proliferation is detected by CCK‐8. Cells were treated with CX‐5461 at 0 nM (DMSO and blank), 25, 50, 75, 100, 500 nM and 1 μM for 1 h and the absorbance at 450 nm was detected at 0, 24 and 48 h, respectively (*n* = 9, Student’s *t*‐test, mean ± SD). (B) Detection of five nucleolar protein expression through western blotting (*n* = 3). (C) The nucleolar region in Figure 4H were magnified and overexposed.

**Figure S7.** Disturbing nucleolar remodelling led to a disordered chromatin landscape. (A) The proportion of images with perinucleolar heterochromatin masses was quantified across different experimental groups. In the D0 group, heterochromatin aggregation around the nucleolus was observed in 13 out of 31 captured images (41.9%). The D2 group showed a reduced incidence, with 6 positive images among 25 captures (24.0%). A progressive decrease was noted in the D4 group, where only 3 of 20 images (15.0%) exhibited this characteristic. The iPSC group demonstrated 2 positive identifications from 11 images (18.2%). Interestingly, the DMSO control group displayed 6 positive observations among 25 images (24.0%), whereas the CX‐5461 treated group showed 7 positive instances out of 16 captured images (43.8%), representing the highest proportion among all groups examined. (B) H3K9me3 protein expression was detected through western blotting (*n* = 3, Student’s *t*‐test, mean ± SD). (C) Immunostaining of nucleolar protein NPM1 and H3K9me3 in the 4F2A MEFs +Dox treated by DMSO (the top row) or CX‐5461 (the bottom row) in D0. Scale bar, 1 μm. (D) 2D intensity histogram is the correlation analysis of green channel (H3K9me3) and red channel (NPM1). Left is DMSO group and right is CX‐5461 group. (E) Peak diagram shows ATAC‐seq of *Fbl*, *Npm1*, *Ubtf*, *Gnl3* and *Ncl*. (F) ATAC‐seq peaks in the DMSO and CX‐5461 group. Each row represents one peak. The colour represents the intensity of chromatin accessibility.

**Figure S8.** Disturbing nucleolar remodelling led to a disordered chromatin landscape. (A) Representative example of auto‐thresholding. The grayscale images represent 8‐bit immunofluorescence image, whereas the red‐highlighted areas indicate threshold‐adjusted selected regions following image processing. (B) Representative example of manual upward adjustment after auto‐thresholding. The grayscale images represent 8‐bit immunofluorescence image, whereas the red‐highlighted areas indicate threshold‐adjusted selected regions following image processing.

**Table S1.** Primer‐detailed sequence of primers in the material method.

**Table S2.** Detailed gene clustering and the first column represents the Ensemble ID of the gene.

**Table S3.** Data used to calculate the nucleolar area ratio.

## Data Availability

The data that support the findings of this study are openly available in GEO at https://www.ncbi.nlm.nih.gov/geo/, reference number GSE232872, GSE227745 and GSE138815. Further information and requests for resources and reagents should be directed to and will be fulfilled by the Lead Contact, Lei Lei (lei086@ems.hrbmu.edu.cn).
