# Supplementary figures and images for: Hypertranscription of rDNA Responsible for Nucleolar Remodelling is a Doorman for Acquiring Pluripotency
Source: Cell Prolif. 2025 May 4;58(10):e70052. doi: 10.1111/cpr.70052 (PMC12508691; doi:10.1111/cpr.70052)

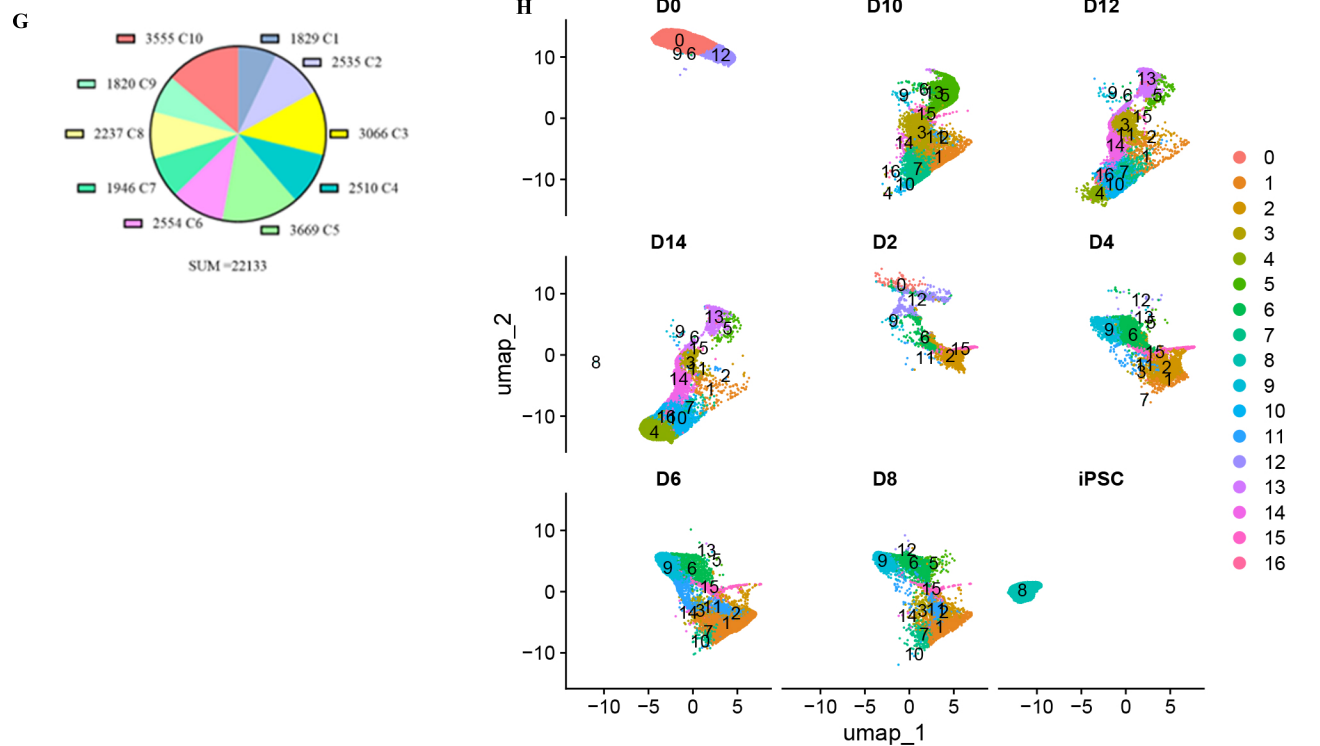

Supplement: Supplementary file 1 — Figure S1. Activation of nucleolar function‐related genes was mainly in the first 2 days of iPSC reprogramming. (A) Unsupervised clustering of mRNA expression in five iPSCs reprogramming stages analysed by RNA‐seq. (B) Principal component analysis of mRNA expression. The same colour represents the same iPSCs reprogramming stage in replicates. (C) Differential gene expression analysis of RNA‐seq data in each reprogramming period. The differential genes among D2 versus D0, D4 versus D2, D6 versus D4 and D8 versus D6 were displayed from left to right. (D) Volcanic map shows differentially expressed genes between D0 and D2. As red plots, significantly upregulated genes. As blue plots, significantly upregulated genes. Ribosome biogenesis related genes are extra noted. (E) The stack bar chart shows the percentage of rDNA transcription related genes (GO#0042790) in each cluster. The most distributed in C10 (red), and followed by clusters 2 and 5 (pink). (F) TPM of DDX21, NCL, Nop16, Polr1a, Polr1e, Wdr36 and Pwp1 in iPSC reprogramming is presented by line chart. (G) The pie chart shows the number of genes in each cluster in Figure 1A. (H) UMAP of single‐cell RNA‐seq labelled by characteristics of cells at different stage of human iPSC reprogramming. [file CPR-58-e70052-s008.pdf]

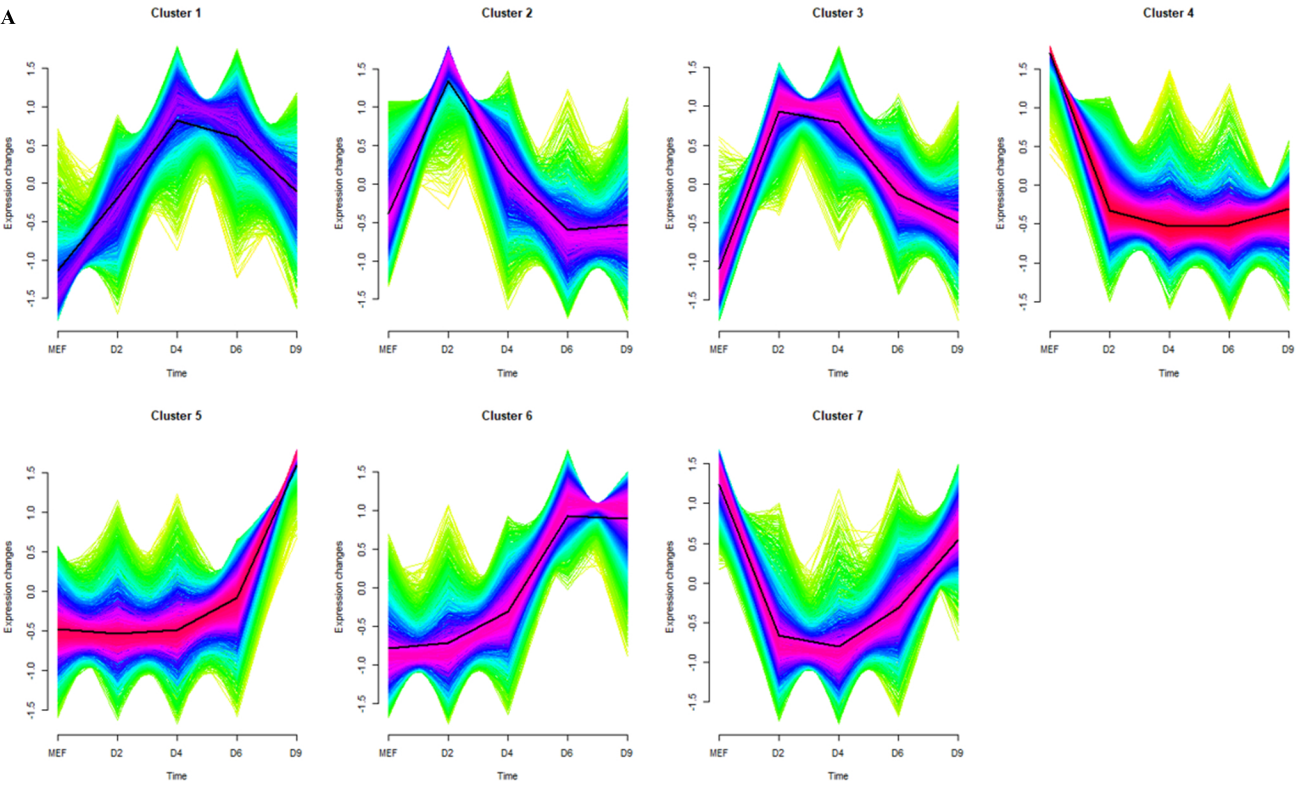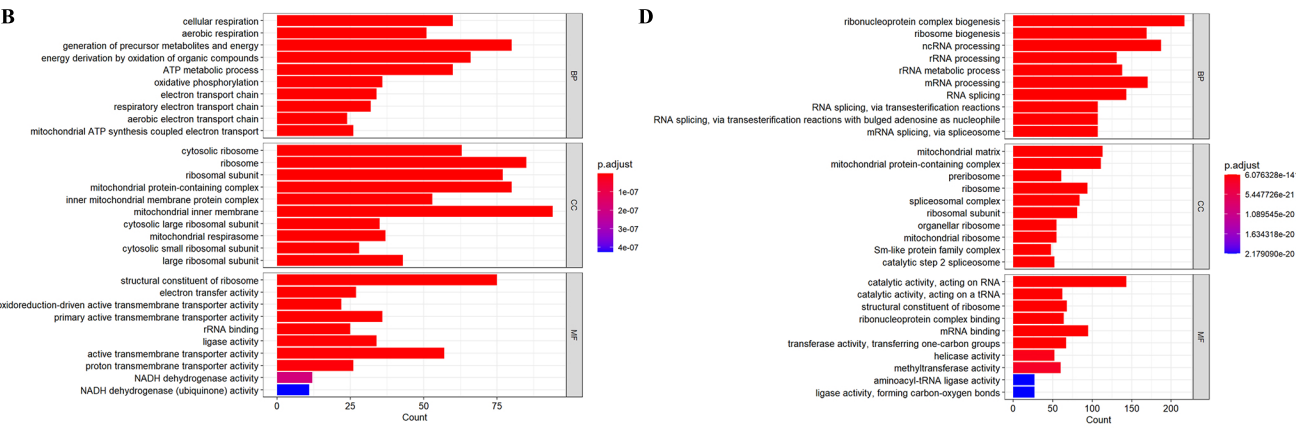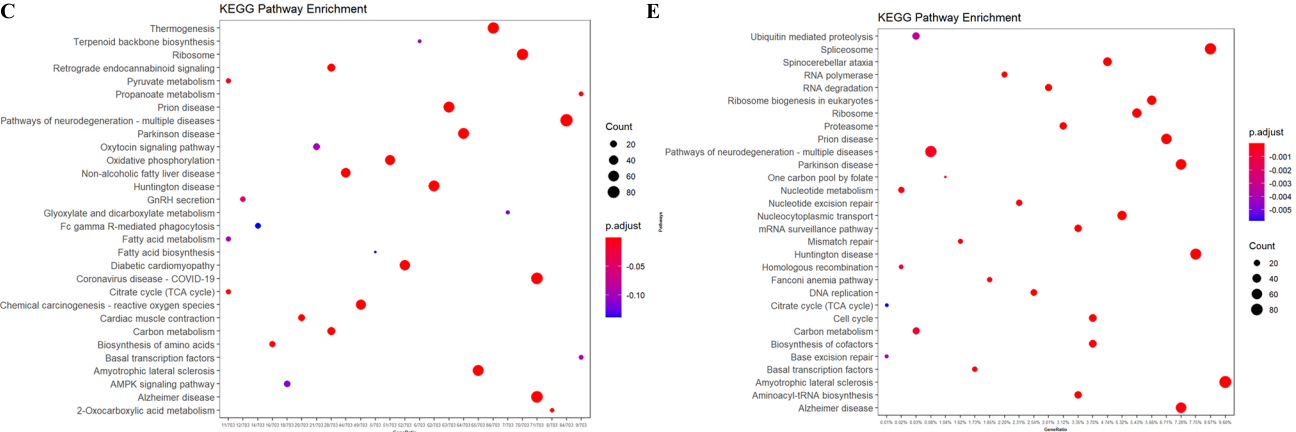

Supplement: Supplementary file 2 — Figure S2. Nucleolus‐related function active in the early stage of iPSCs reprogramming induced by OSKM. (A) Temporal clustering of RNA‐seq date of cells in OSKM iPSCs reprogramming (GSE137001). Fuzzy c‐means clustering identified seven distinct temporal patterns of gene expression in iPSCs reprogramming. The X‐axis represents four points in time for iPSCs reprogramming (D0, D2, D4, D6, D8), whereas the Y‐axis represents log2‐transformed, normalised intensity ratios in each stage. (B, C) GO and KEGG enrichment analysis of cluster 1. (D, E) GO and KEGG enrichment analysis of cluster 3. [file CPR-58-e70052-s010.pdf]

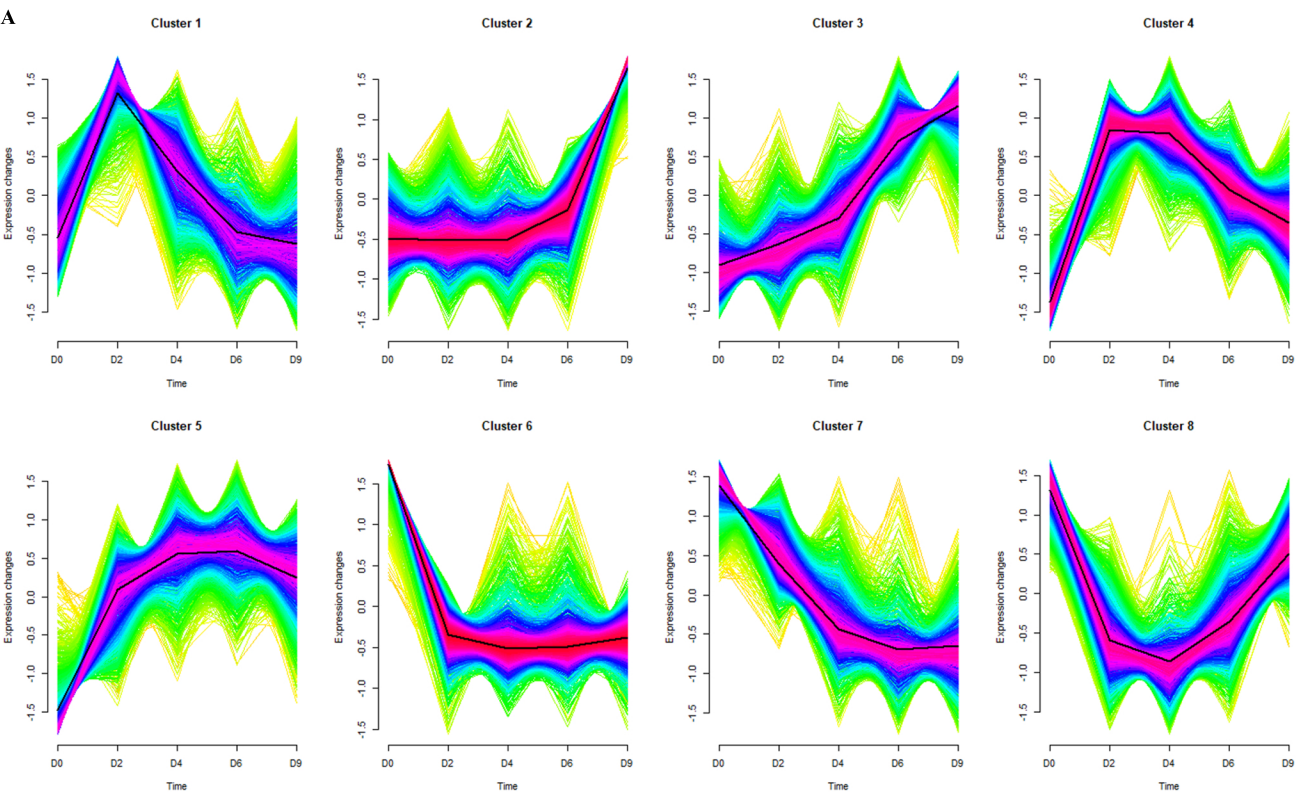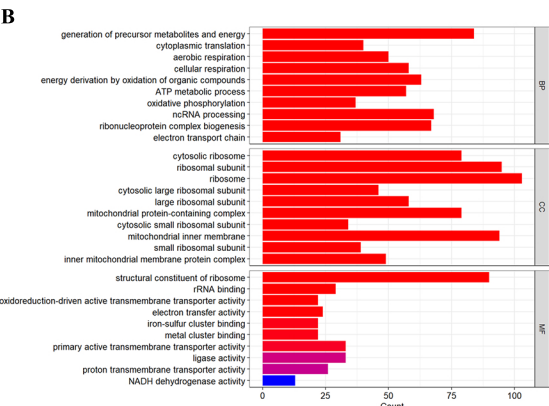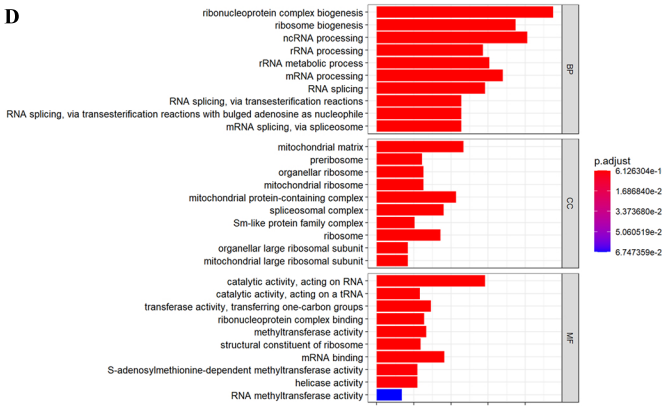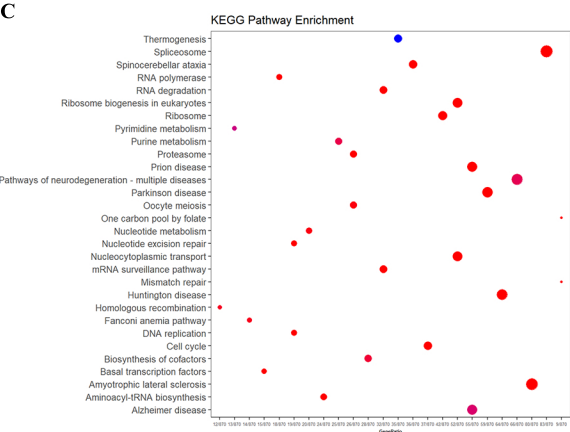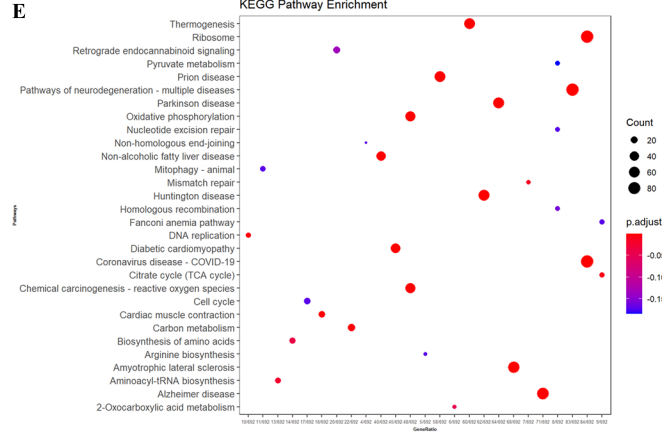

Supplement: Supplementary file 3 — Figure S3. Nucleolus‐related function active in the early stage of iPSCs reprogramming induced by SKM. (A) Temporal clustering of RNA‐seq date of cells in SKM iPSCs reprogramming (GSE137001). Fuzzy c‐means clustering identified eight distinct temporal patterns of gene expression in iPSCs reprogramming. The X‐axis represents four points in time for iPSCs reprogramming (D0, D2, D4, D6, D8), whereas the Y‐axis represents log2‐transformed, normalised intensity ratios in each stage. (B, C) GO and KEGG enrichment analysis of cluster 4. (D, E) GO and KEGG enrichment analysis of cluster 5. [file CPR-58-e70052-s003.pdf]

A

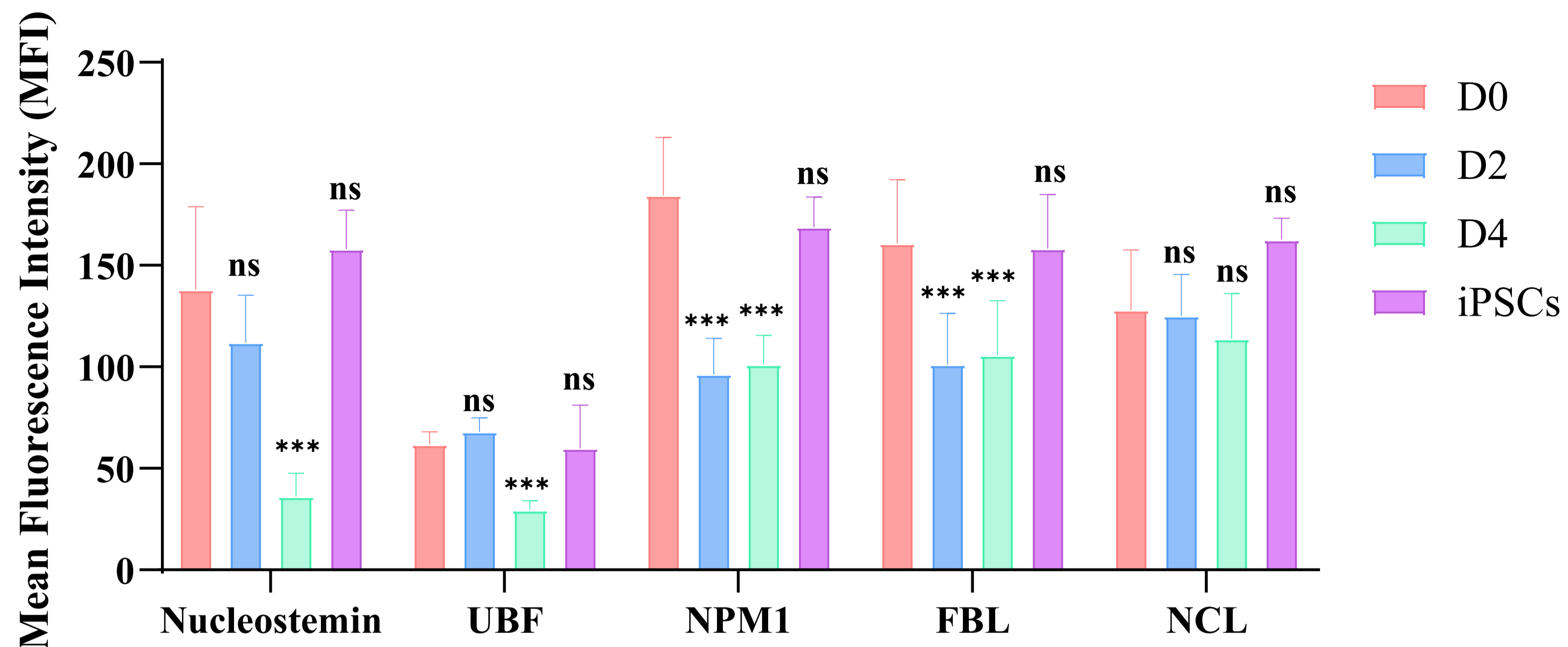

C

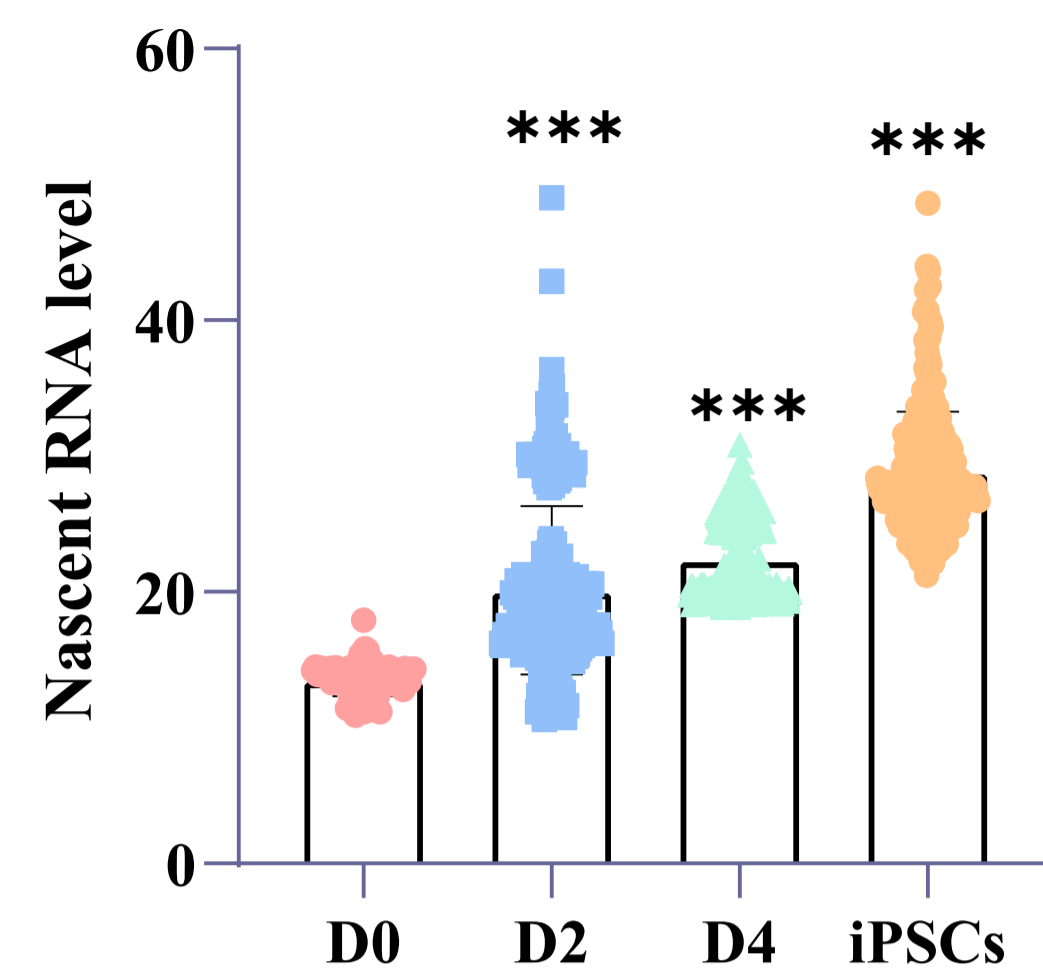

B

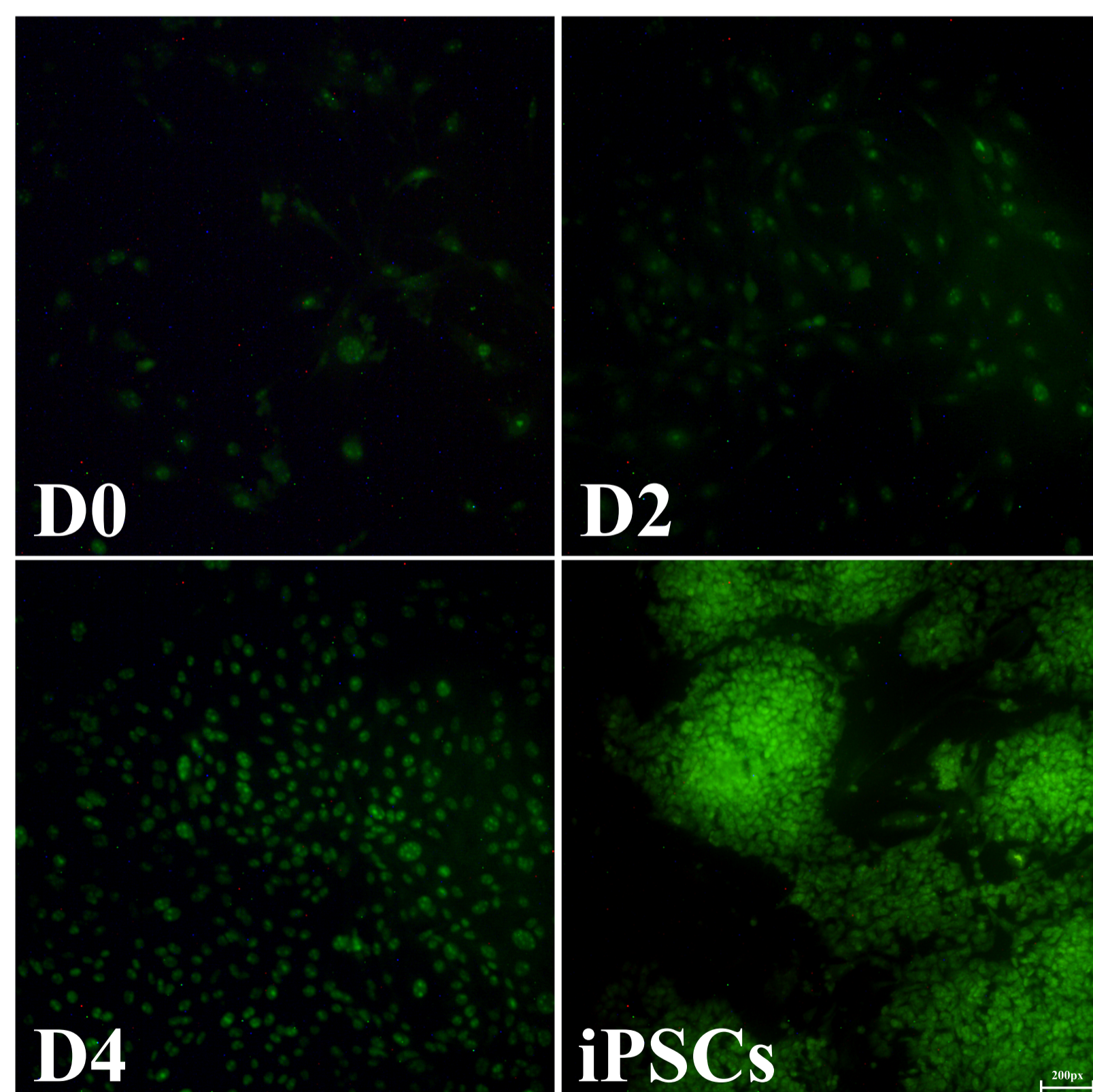

D

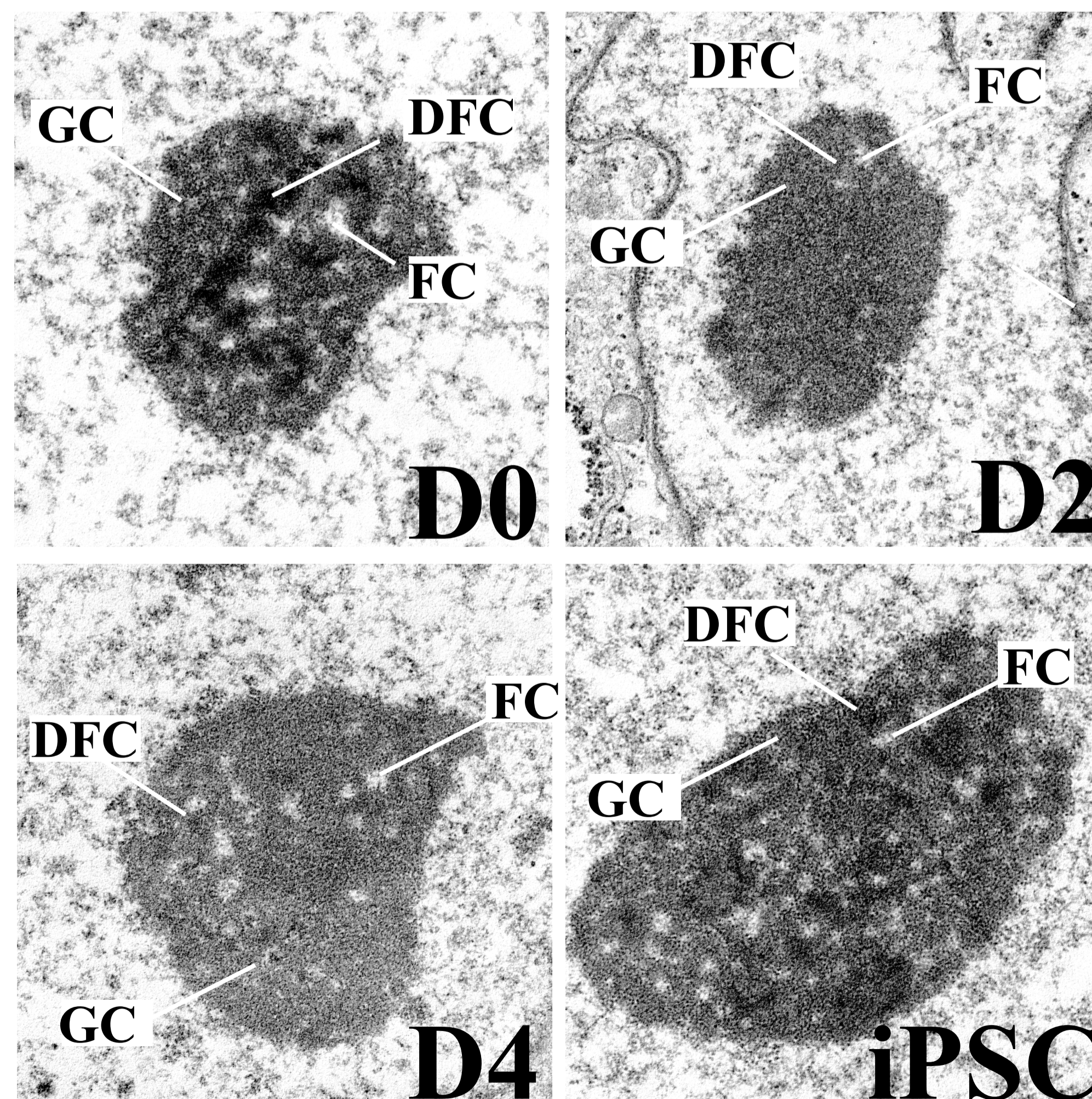

Supplement: Supplementary file 4 — Figure S4. 47S rRNA accumulation originates from enhanced rDNA transcriptional activation within nucleoli. (A) Analysing the immunofluorescence intensity of five nucleolar protein. *p < 0.05, **p < 0.01, ***p < 0.001, Student’s t‐test, mean ± SD. (B, C) 5‐EU staining in the early stage of iPSCs reprogramming (D2, D4), D0 is negative control and iPSCs is positive control. Scale bar, 200 px. The (C) diagram is a statistic of the fluorescence intensity in each cell in the D diagram. (D) The nucleolar region in Figure 2G were magnified and overexposed. [file CPR-58-e70052-s006.pdf]

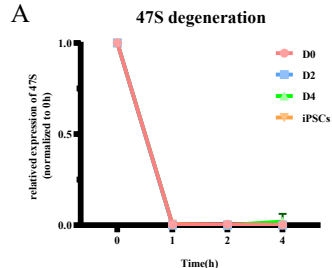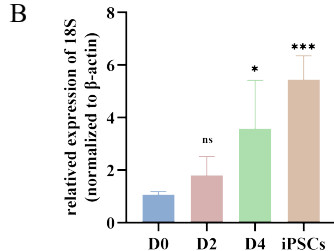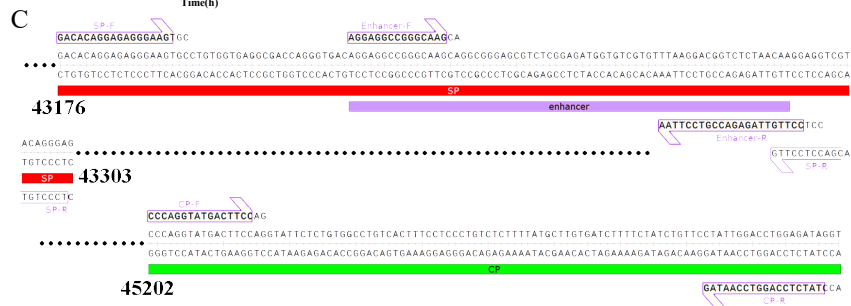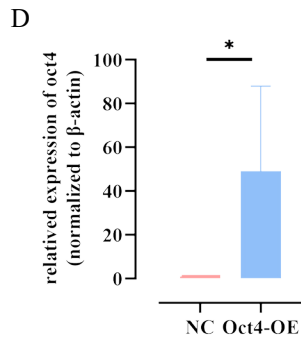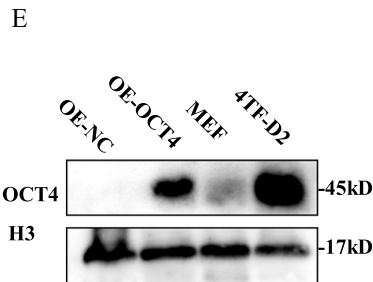

Supplement: Supplementary file 5 — Figure S5. rDNA transcriptional activation and OCT4 can binds to rDNA. (A) At the stages of D0, D2, D4 during reprogramming and the iPSC stage respectively, samples with the same cell quantity were collected at 0, 1, 2 and 4 h after the addition of the actinomycin D (ActD). The content of 47S was detected by qPCR. The expression level at 0 h was set as 1, and the Y‐axis represents the content of 47S at each time point relative to that at 0 h. (B) RT‐qPCR analysis of 18S in the early stage of iPSCs reprogramming (D0, D2, D4) and iPSC, normalised to β‐actin mRNA levels. *p < 0.05, **p < 0.01, ***p < 0.001 (n = 3, Student’s t‐test, mean ± SD). (C) Diagram illustrating the primer of regulatory domain of rDNA. (D) RT‐qPCR analysis of Oct4 mRNA in MEF overexpressed Oct4. *p < 0.05, **p < 0.01, ***p < 0.001 (n = 3, Student’s t‐test, mean ± SD). (E) Detection of Oct4 expression through western blotting. [file CPR-58-e70052-s007.pdf]

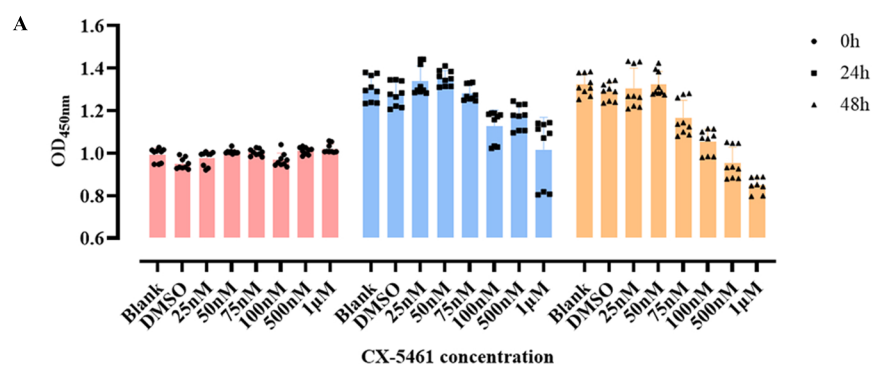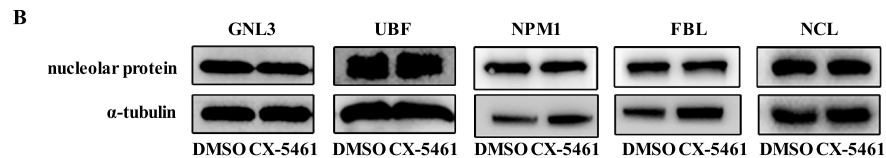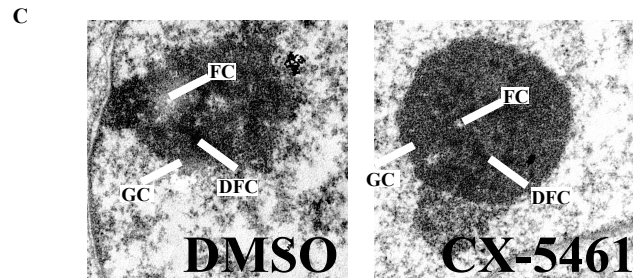

Supplement: Supplementary file 6 — Figure S6. rDNA transcription was crucial to the nucleolar remodelling. (A) Cell proliferation is detected by CCK‐8. Cells were treated with CX‐5461 at 0 nM (DMSO and blank), 25, 50, 75, 100, 500 nM and 1 μM for 1 h and the absorbance at 450 nm was detected at 0, 24 and 48 h, respectively (n = 9, Student’s t‐test, mean ± SD). (B) Detection of five nucleolar protein expression through western blotting (n = 3). (C) The nucleolar region in Figure 4H were magnified and overexposed. [file CPR-58-e70052-s011.pdf]

**A** The proportion of cells with perinuclear heterochromatin masses

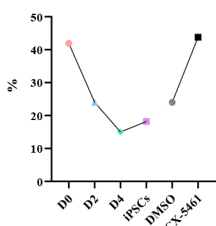

**B** DMSO CX-5461

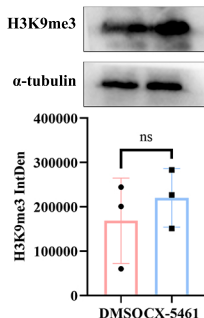

**D** DMSO CX-5461

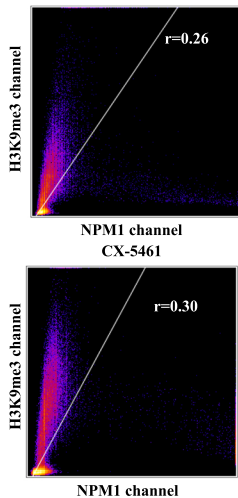

**C**

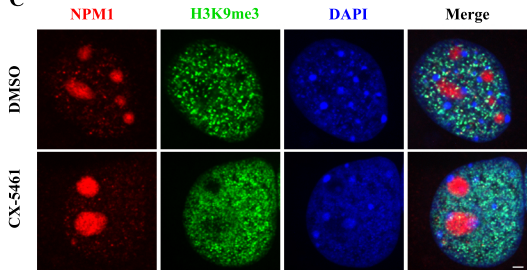

**E**

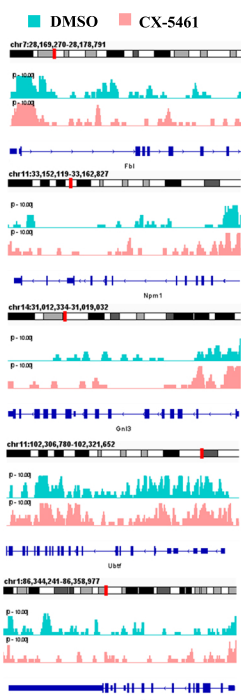

**F**

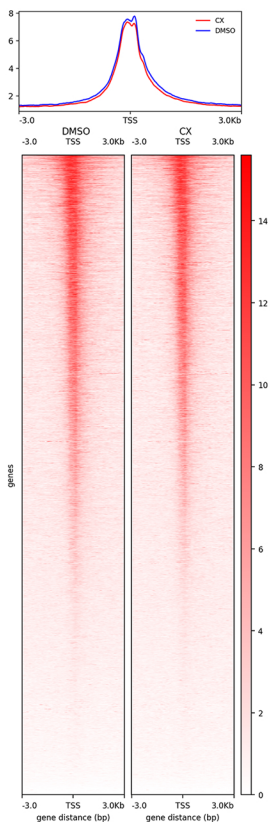

Supplement: Supplementary file 7 — Figure S7. Disturbing nucleolar remodelling led to a disordered chromatin landscape. (A) The proportion of images with perinucleolar heterochromatin masses was quantified across different experimental groups. In the D0 group, heterochromatin aggregation around the nucleolus was observed in 13 out of 31 captured images (41.9%). The D2 group showed a reduced incidence, with 6 positive images among 25 captures (24.0%). A progressive decrease was noted in the D4 group, where only 3 of 20 images (15.0%) exhibited this characteristic. The iPSC group demonstrated 2 positive identifications from 11 images (18.2%). Interestingly, the DMSO control group displayed 6 positive observations among 25 images (24.0%), whereas the CX‐5461 treated group showed 7 positive instances out of 16 captured images (43.8%), representing the highest proportion among all groups examined. (B) H3K9me3 protein expression was detected through western blotting (n = 3, Student’s t‐test, mean ± SD). (C) Immunostaining of nucleolar protein NPM1 and H3K9me3 in the 4F2A MEFs +Dox treated by DMSO (the top row) or CX‐5461 (the bottom row) in D0. Scale bar, 1 μm. (D) 2D intensity histogram is the correlation analysis of green channel (H3K9me3) and red channel (NPM1). Left is DMSO group and right is CX‐5461 group. (E) Peak diagram shows ATAC‐seq of Fbl, Npm1, Ubtf, Gnl3 and Ncl. (F) ATAC‐seq peaks in the DMSO and CX‐5461 group. Each row represents one peak. The colour represents the intensity of chromatin accessibility. [file CPR-58-e70052-s001.pdf]

**A**

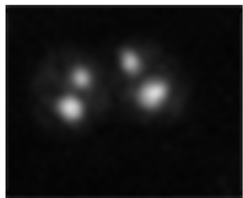

**auto-threshold**

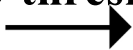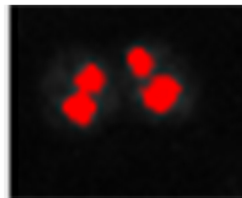

**B**

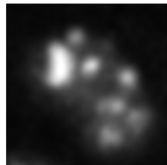

**auto-threshold**

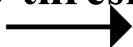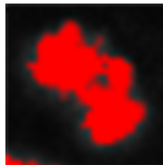

**upward adjustment  
of the threshold**

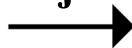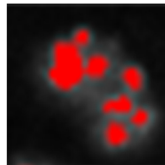

Supplement: Supplementary file 8 — Figure S8. Disturbing nucleolar remodelling led to a disordered chromatin landscape. (A) Representative example of auto‐thresholding. The grayscale images represent 8‐bit immunofluorescence image, whereas the red‐highlighted areas indicate threshold‐adjusted selected regions following image processing. (B) Representative example of manual upward adjustment after auto‐thresholding. The grayscale images represent 8‐bit immunofluorescence image, whereas the red‐highlighted areas indicate threshold‐adjusted selected regions following image processing. [file CPR-58-e70052-s004.pdf]
